# Discovery of Diaryl Ether Substituted Tetrahydrophthalazinones as TbrPDEB1 Inhibitors Following Structure-Based Virtual Screening

**DOI:** 10.3389/fchem.2020.608030

**Published:** 2021-01-21

**Authors:** Erik de Heuvel, Albert J. Kooistra, Ewald Edink, Sjors van Klaveren, Jeffrey Stuijt, Tiffany van der Meer, Payman Sadek, Dorien Mabille, Guy Caljon, Louis Maes, Marco Siderius, Iwan J. P. de Esch, Geert Jan Sterk, Rob Leurs

**Affiliations:** ^1^Division of Medicinal Chemistry, Amsterdam Institute of Molecular and Life Sciences, Vrije Universiteit Amsterdam, Amsterdam, Netherlands; ^2^Laboratory of Microbiology, Parasitology and Hygiene, University of Antwerp, Wilrijk, Belgium

**Keywords:** virtual screening, phosphodiesterase TbrPDEB1, trypanosomiasis, cAMP, tetrahydrophthalazinones, medicinal chemistry

## Abstract

Several members of the 3′,5′-cyclic nucleotide phosphodiesterase (PDE) family play an essential role in cellular processes, which has labeled them as interesting targets for various diseases. The parasitic protozoan *Trypanosoma brucei*, causative agent of human African trypanosomiasis, contains several cyclic AMP specific PDEs from which TbrPDEB1 is validated as a drug target. The recent discovery of selective TbrPDEB1 inhibitors has increased their potential for a novel treatment for this disease. Compounds characterized by a rigid biphenyl tetrahydrophthalazinone core structure were used as starting point for the exploration of novel TbrPDEB1 inhibitors. Using a virtual screening campaign and structure-guided design, diaryl ether substituted phthalazinones were identified as novel TbrPDEB1 inhibitors with IC_50_ values around 1 μM against *T. brucei*. This study provides important structure-activity relationship (SAR) information for the future design of effective parasite-specific PDE inhibitors.

## Introduction

Human African trypanosomiasis, also known as African sleeping sickness, is one of the neglected tropical diseases (NTDs) listed by the WHO and is caused by the protozoan *Trypanosoma brucei* (T.b.) *rhodesiense* and *T.b. gambiense* (Büscher et al., 2017). The majority of drugs that are currently on the market for HAT have been discovered over 30 years ago and have several major disadvantages including severe toxicity, subspecies selectivity, complex administration protocols, and limited clinical efficacy (Babokhov et al., 2013; Eperon et al., 2014; Baker and Welburn, 2018). The first oral drug fexinidazole has recently been approved for HAT and will significantly improve the status of the disease (Deeks, 2019). This new therapy benefits greatly from the ease of administration, but still has some drawbacks including potential relapse and a lower efficacy for late-stage patients compared to the commonly used NECT treatment (De Morais-Teixeira et al., 2019; Pelfrene et al., 2019). In addition, the reported increasing drug resistance could have a detrimental effect on the already limited arsenal of antiprotozoal drugs (Munday et al., 2015; De Koning, 2017). The number of reported cases is slowly decreasing as a result of active screening in endemic regions, still an estimated 65 million people are at risk of infection (World Health Organization, 2020). HAT has a history that is characterized by reoccurring epidemics and new control strategies and safer drugs are therefore still a necessity to eradicate this fatal disease (Brun et al., 2010; Büscher et al., 2017; Baker and Welburn, 2018).

The family of 3′,5′-cyclic nucleotide phosphodiesterases (PDEs) are involved in various essential regulatory processes in many different organisms making them interesting drug targets. The human 3′,5′-cyclic nucleotide phosphodiesterases (hPDE) have been extensively studied as drug targets for a broad range of diseases, including COPD, heart failure, and erectile dysfunction (Packer et al., 1991; Boolell et al., 1996; Hatzelmann and Schudt, 2001; Calverley et al., 2009). The *T. brucei* 3′,5'-cyclic nucleotide phosphodiesterases B1 (TbrPDEB1) and TbrPDEB2 have previously been identified as potential new targets for the treatment of HAT as, in contrast to the other TbrPDE enzymes, they are essential for parasite virulence (Oberholzer et al., 2007). Simultaneous reduction in expression of TbrPDEB1 and TbrPDEB2 with siRNA resulted in distortions of the cell cycle and eventually cell death (Kunz et al., 2006; Oberholzer et al., 2007). The potential of TbrPDEB1 and TbrPDEB2 as drug targets against HAT was further demonstrated *in vivo* as siRNA-mediated gene silencing in mice prevented parasitemia and finally resulted in animal survival after parasite infection (Oberholzer et al., 2007). Simultaneous inhibition of both isoforms by small molecule inhibitors is conceived possible because of high structural similarity between both paralogues (88% structural identity of the catalytic domain), resulting in a high degree of equipotency as reported for NPD-001 (IC_50_ TbrPDEB1: 12.0 nM; IC_50_ TBrPDEB2: 12.4 nM) (De Koning et al., 2012; Orrling et al., 2012; Veerman et al., 2016).

**Figure 1:**
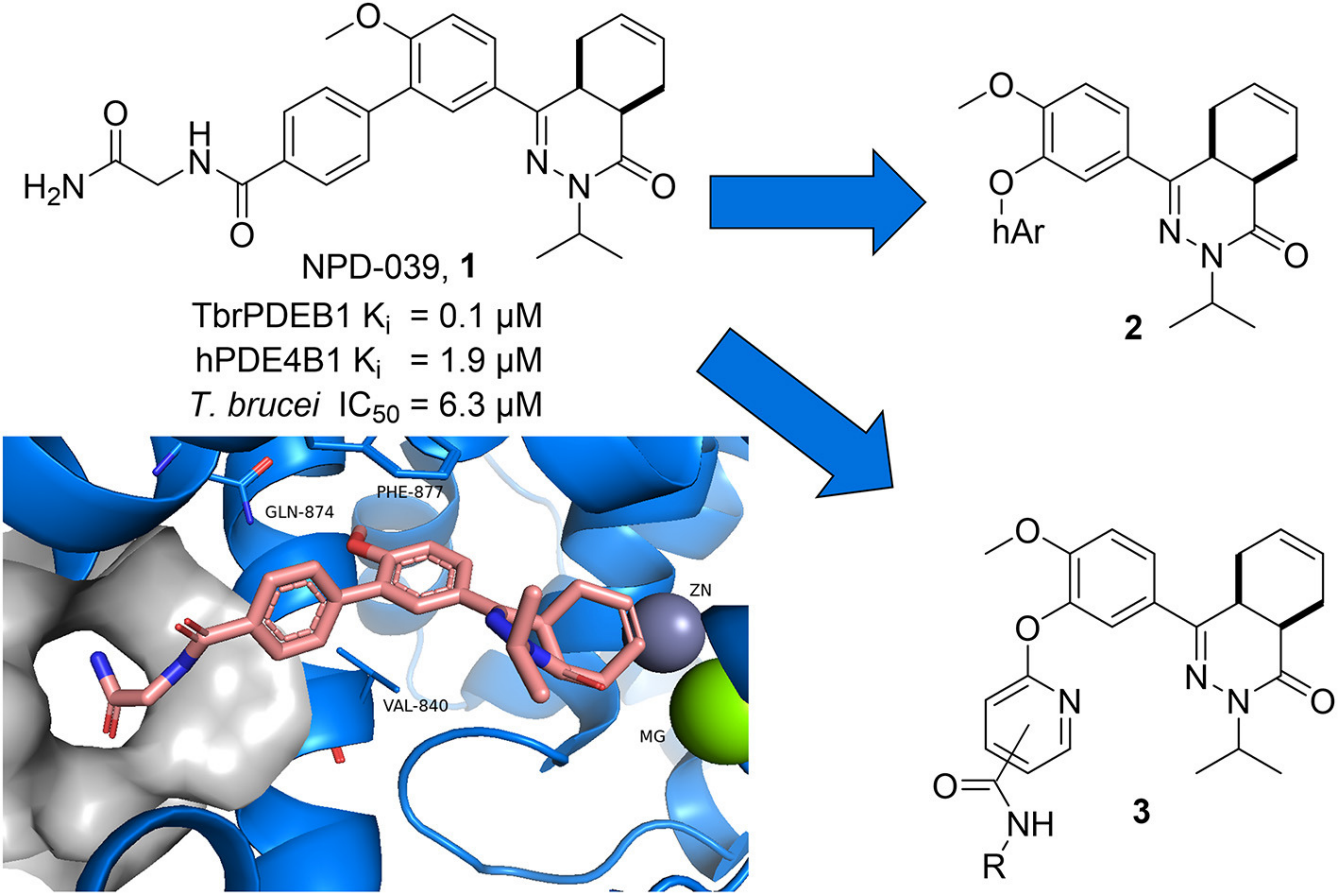
Design ideas based on reported biphenyl phthalazinone NPD-039 (1) based on virtual screening using the co-crystal structure of 1 (PDB: 5L8C) and heteroaryl chlorides (2) and structure-guided design (3).

Recently, a first series of molecules with selectivity for TbrPDEB1 over hPDE4 was reported by repurposing a tetrahydrophthalazinone scaffold that was originally developed as hPDE4 inhibitor (Van Der Mey et al., 2001a,b; Blaazer et al., 2018). Potency and selectivity over hPDE4 was obtained by addressing a parasite-specific pocket (P-pocket) in the substrate-binding site of TbrPDEB1 with a rigid biphenyl glycinamide installed on the tetrahydrophthalazinone (NPD-039, shown in Figure 1) (Jansen et al., 2013; Blaazer et al., 2018). NPD-039 (**1**) displays high nanomolar potency for TbrPDEB1 (K_i_ = 0.1 μM) with more than 10-fold selectivity over hPDE4 (K_i_ = 1.9 μM) with the glycinamide tail occupying the P-pocket in the crystal structure of **1** in the catalytic domain of TbrPDEB1 (Blaazer et al., 2018). Unfortunately, **1** shows a reduced efficacy against *T. brucei in vitro* (IC_50_ = 6.3 μM) and its development as trypanocidal was therefore halted (Blaazer et al., 2018).

In the present study, we describe one of our efforts to improve on **1** by introducing flexibility into the vector that directs to the P-pocket using a diaryl ether function. Two different design strategies were applied in parallel. Firstly, computer-aided drug design using the structure of NPD-039 co-crystalized in TbrPDEB1 (Figure 1, PDB: 5L8C) and commercially available heteroaromatic moieties (hAr, **2**) provided a selection of virtual hits for synthesis to explore accessibility of various aromatic structures in the active site of TbrPDEB1. Secondly, the pyrimidyl group in **3** was decorated with a selection of amide functionalities based on observations in previously reported studies to explore the directionality toward the P-pocket (Blaazer et al., 2018; De Heuvel et al., 2019b). Both compound classes were synthesized and tested to explore the interaction with TbrPDEB1 and their *in vitro* efficacy against *T. brucei*.

## Materials and Methods

### Phosphodiesterase Activity Assays

The TbrPDEB1 catalytic domain phosphodiesterase activity assays were conducted based on a method reported by Sijm et al. (2019) with minor adaptations (Sijm et al., 2019). The PDELight™ HTS cAMP phosphodiesterase Kit (Lonza, Walkersville, USA) was performed at 25°C in non-binding, low volume 384-well plates (Corning, Kennebunk, ME, USA). PDE activity measurements (TbrPDEB1_CD; K_m_ 3.45 μM, hPDE4B_CD; K_m_ 13.89 μM) were made in “stimulation buffer” (50 mM Hepes, 100 mM NaCl, 10 mM MgCl_2_, 0.5 mM EDTA, 0.05mg/mL BSA, pH 7.5). Single concentration measurements were made at 10 μM inhibitor concentration (triplo measurements/assay, n = 2). Dose-response curves were made in the range 100 μM−10 pM (triplo measurements/assay, *n* = 3). Compounds were diluted in DMSO (final in-test concentration 1%). Inhibitor dilutions (2.5 μL) were transferred to the 384-well plates, 2.5 μL of PDE in stimulation buffer was added and mixed, 5 μL of cAMP (at 2 × K_m_ up to 20 μM) added and the assay mixture was incubated for 20 min at 300 rpm. The reaction was terminated by the addition of 5 μL of Lonza Stop Buffer supplemented with 10 μM of NPD-001. Then, 5 μL of Lonza Detection reagent (diluted to 80% with reaction buffer) was added and the reaction incubated for 10 min at 300 rpm. Luminescence was read with a Victor3 luminometer using a 0.1 s/well program.

RLUs were measured in comparison to the DMSO-only control, NPD-001 always was taken along as positive control as a PDE inhibitor. The K_i_ values of the inhibitors analyzed are represented as the mean of at least three independent experiments with the associated standard error of the mean (S.E.M.). Due to solubility issues, we were not able to determine full dose-response curves for all compounds; K_i_ values for such inhibitors were obtained by curve fitting (Graphpad Prism 7.0) and the assumption of full inhibition to a level of inhibition by NPD-001.

### Phenotypic Cellular Assays

The phenotypic cellular assays were conducted as previously reported by Blaazer et al. (Blaazer et al., 2018). For the cellular assays, reference drugs as positive controls were suramin (Sigma-Aldrich, Germany) for *T. brucei* (IC_50_ = 0.04 ± 0.02 μM, *n* = 5) and tamoxifen (Sigma-Aldrich, Germany) for MRC-5 cells (CC_50_ = 10 ± 2.1 μM, *n* = 5). All compounds were tested at five concentrations (64, 16, 4, 1, and 0.25 μM) to establish a full dose-titration and determination of the IC_50_ and CC_50_, data are represented as the mean of duplicate experiments ± S.E.M. The final in-test concentration of DMSO did not exceed 0.5%.

For the antitrypanosomal assay, *T.b. brucei* Squib-427 strain (suramin-sensitive) was cultured at 37°C and 5% CO_2_ in HMI-9 medium supplemented with 10% fetal calf serum (FCS). Approximately 1.5 × 10^4^ trypomastigotes were added to each well and parasite growth was assessed after 72 h at 37°C by adding resazurin. Viability was assessed fluorimetrically 24 h after addition of resazurin. Fluorescence was measured (excitation 550 nm, emission 590 nm) and the results were expressed as percentage reduction in viability compared to control.

For the cellular cytotoxicity assay, MRC-5 SV2 cells, originally from a human diploid lung cell line, were cultivated in MEM supplemented with L-glutamine (20 mM), 16.5 mM sodium hydrogen carbonate and 5% FCS. For the assay, 10^4^ MRC-5 cells/well were seeded onto the test plates containing the pre-diluted sample and incubated at 37°C and 5% CO_2_ for 72 h. Cell viability was assessed fluorimetrically 4 h after the addition of resazurin. Fluorescence was measured (excitation 550 nm, emission 590 nm) and the results were expressed as percentage reduction in cell viability compared to controls.

### Chemistry

All reagents and solvents were obtained from commercial suppliers and were used as received. All reactions were magnetically stirred and carried out under an inert atmosphere. Reaction progress was monitored using thin-layer chromatography (TLC) and LC-MS analysis. LC-MS analysis was performed on a Shimadzu LC-20AD liquid chromatograph pump system, equipped with an Xbridge (C18) 5 μm column (50, 4.6 mm), connected to a Shimadzu SPD-M20A diode array detector, and MS detection using a Shimadzu LC-MS-2010EV mass spectrometer. The LC-MS conditions were as follows: solvent A (water with 0.1% formic acid) and solvent B (MeCN with 0.1% formic acid), flow rate of 1.0 mL/min, start 5% B, linear gradient to 90% B in 4.5 min, then 1.5 min at 90% B, then linear gradient to 5% B in 0.5 min, then 1.5 min at 5% B; total run time of 8 min. Silica gel column chromatography was carried out with automatic purification systems using the indicated eluent. Reversed phase column purification was performed on the Grace Davison iES system with C18 cartridges (60 Å, 40 μm) using the indicated eluent. Nuclear magnetic resonance (NMR) spectra were recorded as indicated on a Bruker Avance 500 (500 MHz for ^1^H and 125.8 MHz for ^13^C) instrument equipped with a Bruker CryoPlatform, or on a Bruker DMX300 (300 MHz for ^1^H) or a Bruker Biospin (400 MHz for ^1^H). Chemical shifts (δ in ppm) and coupling constants (*J* in Hz) are reported with residual solvent as internal standard (δ ^1^H-NMR: CDCl_3_ 7.26; DMSO-*d*_6_ 2.50; δ ^13^C-NMR: CDCl_3_ 77.16; DMSO-*d*_6_ 39.52). Abbreviations used for ^1^H-NMR descriptions are as follows: s = singlet, d = doublet, t = triplet, q = quintet, hept = heptet, dd = doublet of doublets, dt = doublet of triplets, tt = triplet of triplets, m = multiplet, app d = apparent doublet, br = broad signal. Exact mass measurements (HRMS) were performed on a Bruker micrOTOF-Q instrument with electrospray ionization (ESI): in positive ion mode and a capillary potential of 4,500 V. Microwave reactions were carried out in a Biotage Initiator^+^ using sealed microwave vials. Systematic names for molecules were generated with the ChemBioDraw Ultra 14.0.0.117 (PerkinElmer, Inc.). The reported yields refer to isolated pure products and are not optimized. The purity, reported as the LC peak area % at 254 nm, of all final compounds was ≥95% based on LC-MS. All compounds are isolated as a racemic mixture of *cis*-enantiomers. A detailed overview of the synthetic procedures can be found in the Supplementary Information (SI) in Supplementary Material.

## Results and Discussion

### Virtual Screening

**Figure 2:**
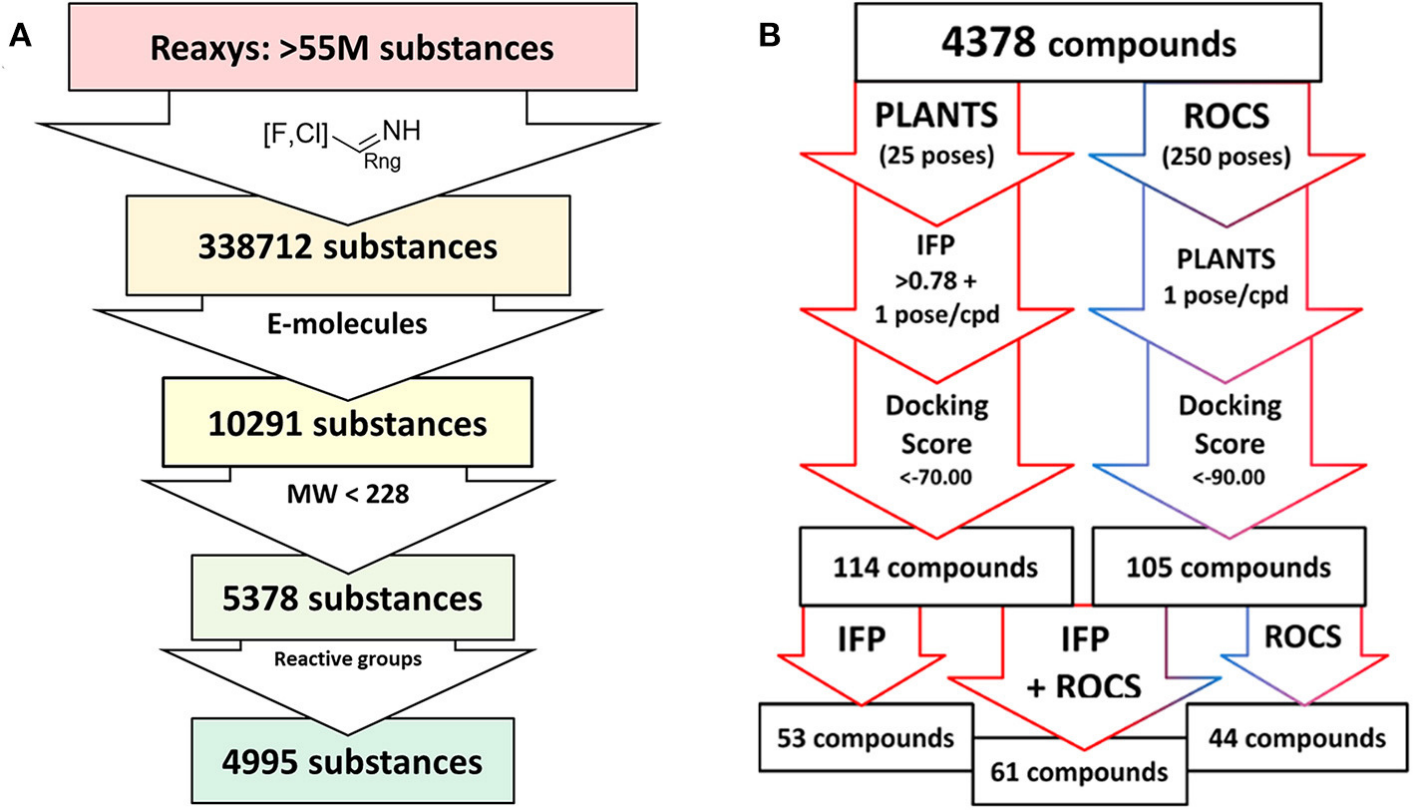
Schematic overview of the virtual screening. **(A)** Extraction of commercially available heteroaromatic chlorides and fluorides from Reaxys. Every arrow indicates a filter step based on substructure, commercial availability, molecular weight, and a preliminary filter on reactive functionalities. **(B)** Schematic overview of the docking process based on PLANTS/IFP protocol or ROCS/PLANTS protocol of all compounds obtained from the Reaxys search.

The compound dataset for the virtual screening was based on commercially available heteroaryl chlorides which were combined with the core phenyltetrahydrophthalazinone scaffold using the MOE Combinatorial Library module (Figure 2). Reaxys was used to search for commercially available building blocks to use in a straightforward nucleophilic aromatic substitution reaction with a phenol tetrahydrophthalazinone. The Reaxys search was focused on commercially available (via E-molecules) heteroaromatic chlorides with a molecular weight <228 Da to design a library of compounds with a maximum molecular weight of 500 Da. The combinatorial library consisted of almost 5,000 compounds which were docked in the crystal structure of NPD-039 (**1**, PDB: 5L8C) using two different methods. Firstly, all compounds were docked using PLANTS and scored based on the similarity of the interaction fingerprint (IFP) compared with NPD-039. A high IFP similarity with NPD-039 suggests a similar binding mode and a higher probability of having similar affinity. All compounds were also scored using the overall docking score. Using a combination of scoring criteria (an IFP similarity >0.78 and a docking score <70) resulted in 114 selected compounds. In a second virtual screening approach, all computation library compounds were compared to the binding pose of NPD-039 in TbrPDEB1 using ROCS. The best scoring pose per compound was docked in the crystal structure of TbrPDEB1 using PLANTS and the compounds with a docking score <90 were selected, resulting in 105 compounds.

**Figure 3:**
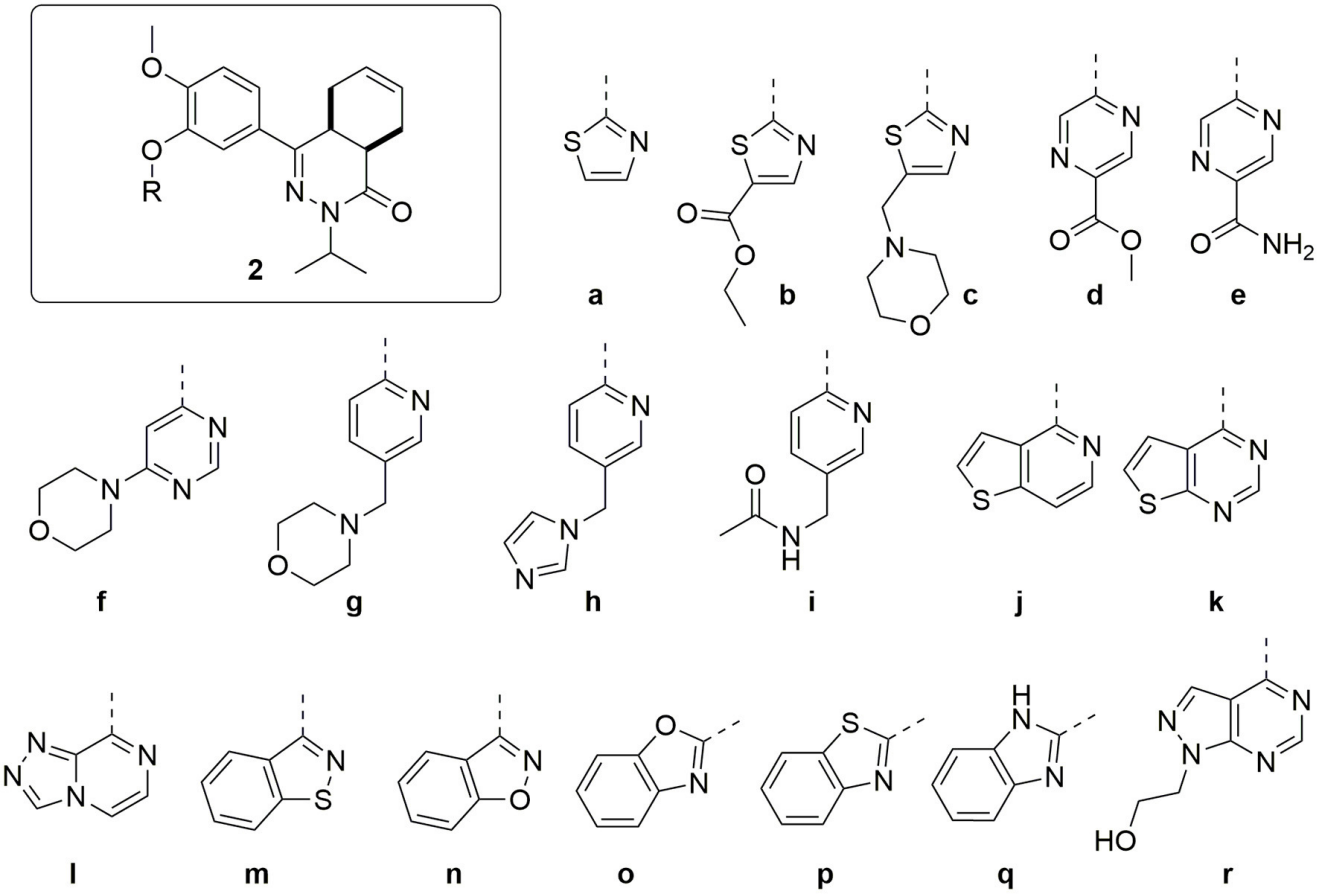
Structures of the selected hits from the virtual screening.

**Figure 4:**
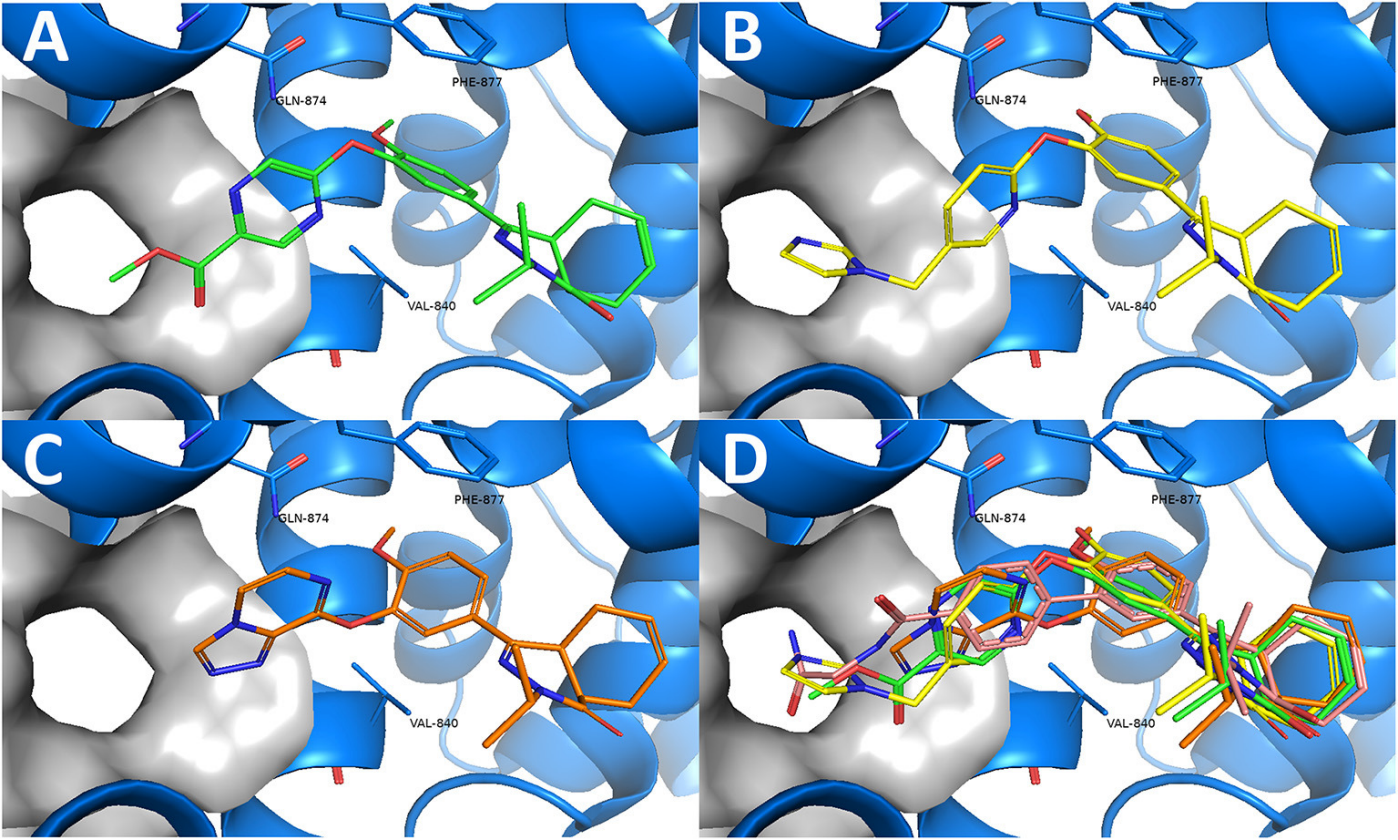
Examples of docked structures in the co-crystal structure of 1 in TbrPDEB1 (PDB: 5L8C). The P-pocket is indicated by the gray surface. **(A)** The docking pose of pyrazine 2d (green). **(B)** The docking pose of 2h (yellow). **(C)** The docking pose of 2l (orange). **(D)** Overlay of the different docking poses of 2d, 2h, and 2l with reference compound 1 (salmon).

Combining the hit sets from both virtual screening strategies (ROCS and PLANTS) resulted in 158 unique compounds that were visually inspected for their synthetic feasibility and binding mode in the crystal structure, resulting in a selection of 45 compounds (see SI). The compounds were divided into four clusters: 5-membered ring structures, 6-membered ring structures, and fused 5- and 6-membered ring structures (5-ring linked or 6-ring linked). Several representatives from every cluster were selected for synthesis to assure the presence of every ring size in the final set of compounds (Figure 3). For some of the hits, reagents turned out to be more difficult to obtain or expensive; in those cases a more readily available building block to represent the same cluster was used. However, the replacement often resulted in the selection of simplified and rigid building blocks that lack flexible substituents that can penetrate the P-pocket as observed for **1** (Figure 1). It was hypothesized that favorable binding to TbrPDEB1 could be obtained by introducing rigid aromatic systems, as previously observed for the biphenyl series (Blaazer et al., 2018). The docking pose of these more rigid compounds (**2a**, **2j-q**, and to lesser extent conjugated esters **2b**, **2d**, and amide **2e**) showed good directionality toward the P-pocket, as illustrated by the docking pose of **2d** (Figure 4A), but did not address or interact with residues in the P-pocket. Nevertheless, the docking pose of these compounds provide essential information for possible future modifications sites. The docked hits containing a flexible substituent (**2c**, **2f-i**, **2q**) showed good occupation of the P-pocket, as illustrated by the docking pose of **2h** (Figure 4B). The introduction of the ether bond between the aromatic functionalities causes a slight rotation of the anisole in the core scaffold. In most cases, the ether bond is rotated toward the phenylalanine of the hydrophobic clamp, while for several of the larger fused 5- and 6-membered rings (**2j**, **2l**, **2m**, and **2n**) the ether linker is rotated toward the valine (Figure 4C). Although the spatial filling of the linker is divergent from the phenyl linker of **1** (Figure 4D), the occupancy of substituents is similar to the of the glycinamide tail of **1**. A detailed overview of the individual docking poses of **2a-r** can be found in the supporting information.

### Chemistry

**Scheme 1:**
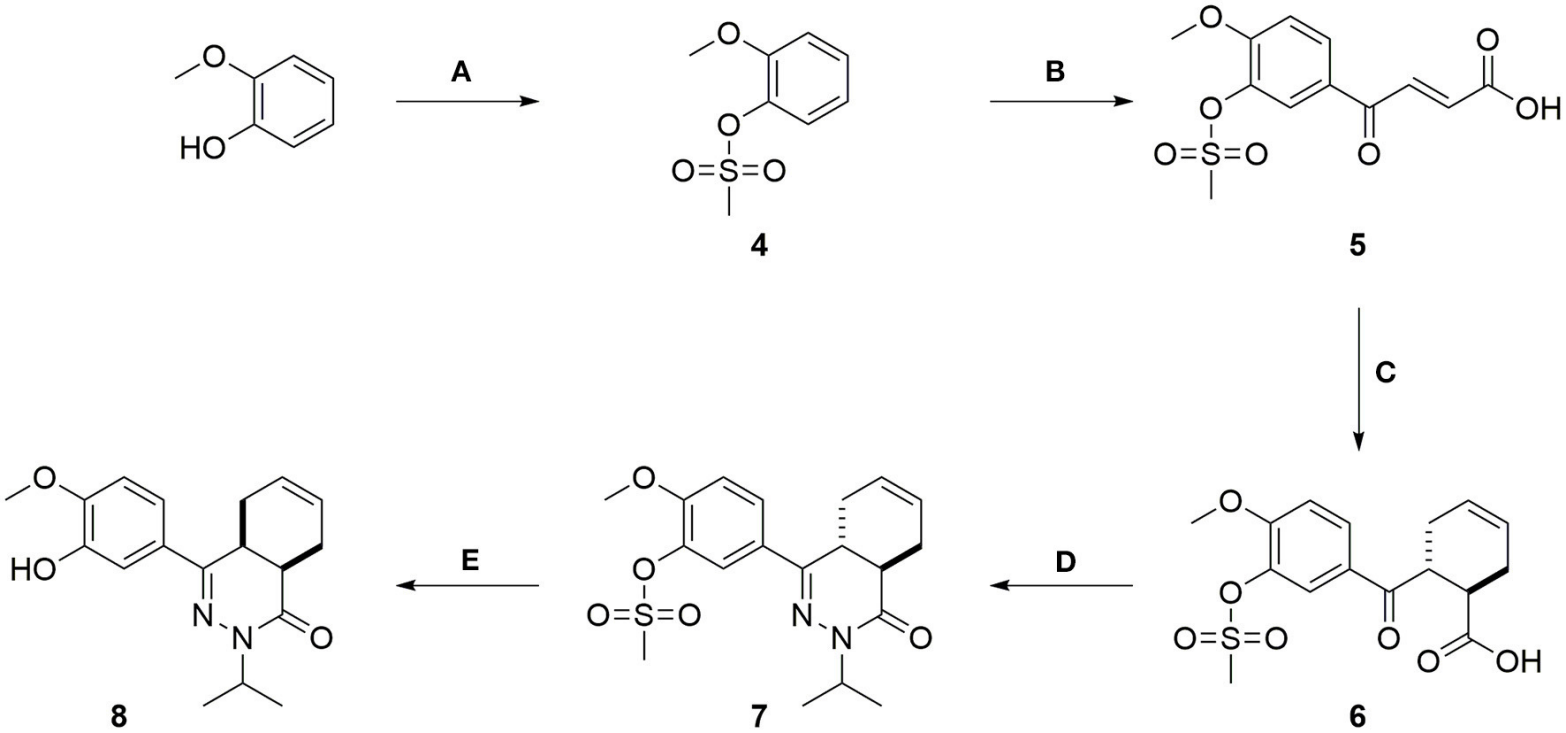
**(A)** Mesyl chloride, Et_3_N, DCM, rt, 1 h, 78%; **(B)** Maleic anhydride, AlCl_3_, DCM, rt, 5 h, 40%; **(C)** Buta-1,3-diene, THF, 140°C, MW, 1.5 h, 92%; **(D)** Isopropylhydrazine.HCl, Cs_2_CO_3_, EtOH, 100°C, MW, 6 h, 75%; **(E)** NaOH, H_2_O, THF, MeOH, 50°C, 2 h, 64%.

The synthesis of the compounds started with mesylation of guaiacol using mesyl chloride (Scheme 1). Mesylate **4** was used in a Friedel-Crafts acylation with maleic anhydride to obtain carboxylic acid **5**. Full isomerization toward the *E*-isomer was observed during the reaction. The *trans*-cyclohexene moiety of carboxylic acid **6** was installed using a Diels-Alder reaction with 1,3-butadiene, which was then used in a condensation reaction with isopropyl hydrazine to obtain tetrahydrophthalazinone **7**. The mesylate group was partially removed during the condensation reaction due to the basic conditions and a mixture of products was obtained. The mesylate group was completely removed by subjecting the mixture to a solution of NaOH in MeOH/THF. As shown for similar phenyl tetrahydrophthalazinones, *trans*-cyclohexene isomerizes to the *cis*-cyclohexene under basic conditions (De Heuvel et al., 2019a). The *cis*-conformation was confirmed by a strong NOE-coupling between the two bridgehead protons of tetrahydrophthalazinone **8**. The obtained phenol tetrahydrophthalazinone **8** was used as a building block for the synthesis of the various target compounds.

**Scheme 2:**
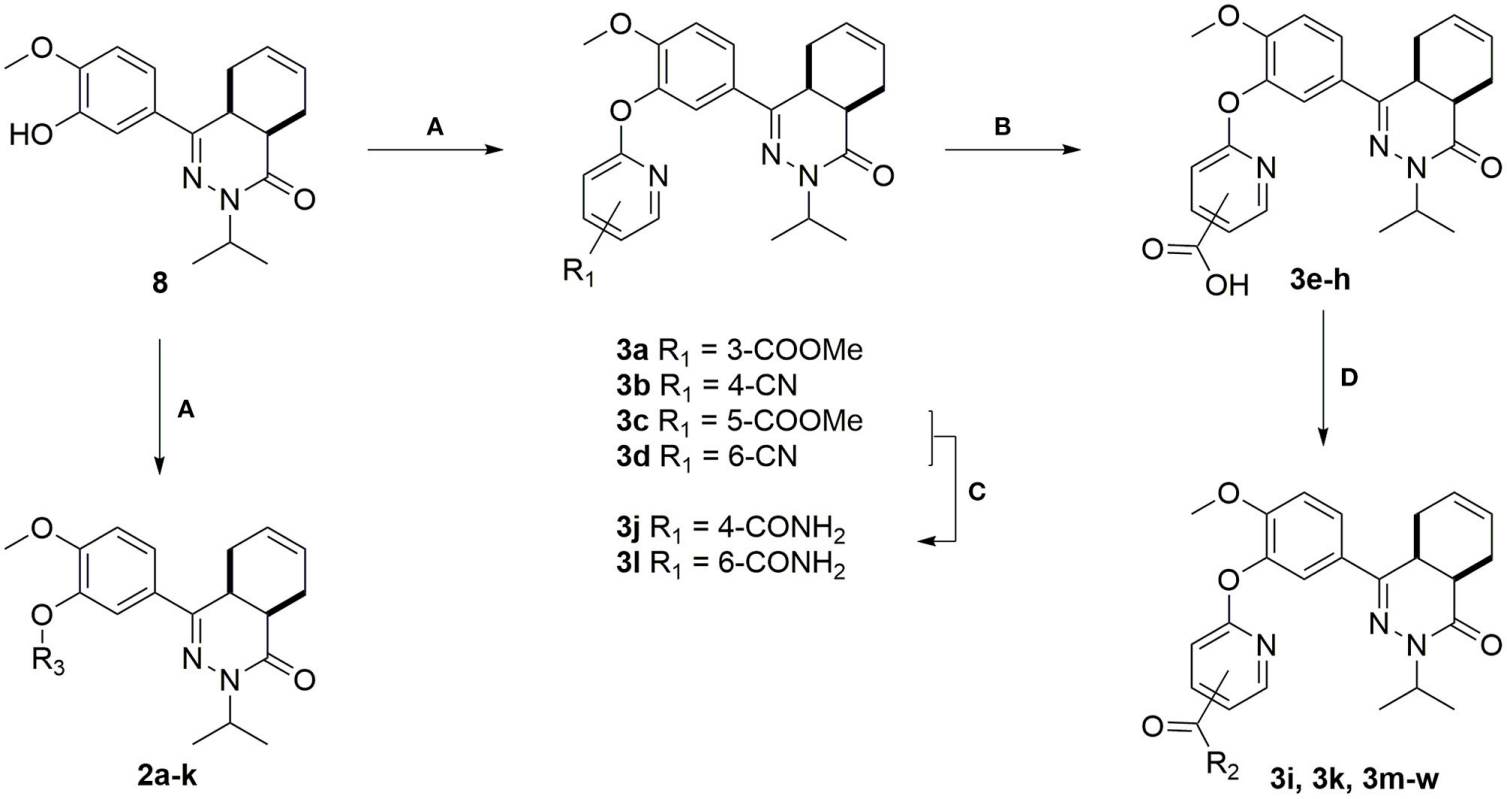
**(A)** Corresponding aryl chloride, Cs_2_CO_3_, DMF, 100°C, 1–16 h, 23–82%; **(B)** NaOH, H_2_O, MeOH, rt, overnight, 71–99%; **(C)** 30% aq. H_2_O_2_, K_2_CO_3_, DMSO, 0 °C, 10 min, 3j: 71%, 3l: 99%; **(D)** Corresponding amine, EDC.HCl, HOBt.H_2_O, DCM, rt, 16–72 h, 28–81%.

The heteroaromatic ring systems were installed in the tetrahydrophthalazinone core structure using a nucleophilic aromatic substitution reaction at higher temperatures (Scheme 2). The reactivity of the various aromatic chlorides differed significantly, leading to varying reaction times and yields. Unfortunately, the synthesis of **2g**, **2i**, **2m-o**, **2q** was unsuccessful due to observed side reactions or instability of the starting material under the reaction conditions. The synthesis of 4- and 6-methyl ester functionalized chloropyridines did not provide the desired intermediates. Therefore, the methyl ester was replaced by a nitrile (**3b** and **3d**) to provide a handle for further modifications. Both methyl esters (**3a** and **3c**) and nitriles (**3b** and **3d**) were successfully hydrolyzed with NaOH to obtain carboxylic acids **3e-h**. Furthermore, the nitriles were used in a Radziszewski reaction to quickly and efficiently obtain the carboxamides **3j** and **3l**. All other tail groups (**3i**, **3k**, and **3m-w**) were installed in an amide coupling using EDC/HOBt. Unfortunately, the synthesis and functionalization of the 3-position on the pyridine using glycinamide was troublesome and unsuccessful.

### Biochemical Activity

All compounds were initially tested for their biochemical activity against TbrPDEB1 in a single point assay at 10 μM. All of the 5-membered and 6-membered rings (**2a-f** and **2h**) from the virtual screening hits showed low to moderate inhibition of TbrPDEB1 at this concentration (Table 1), but the larger fused 5- and 6-membered rings (**2j-l** and **2p**) showed no inhibition at 10 μM. The observation of deviant binding poses for this cluster in the virtual screening and the absence of activity suggests that these linkers are too bulky to fit in the limited space toward the P-pocket. The best results were observed for pyrazine **2d** and pyridine **2h**, both having about 50% inhibition at 10 μM.

**Table 1:** Single point activities of the virtual screening hits against TbrPDEB1 at 10 μM.

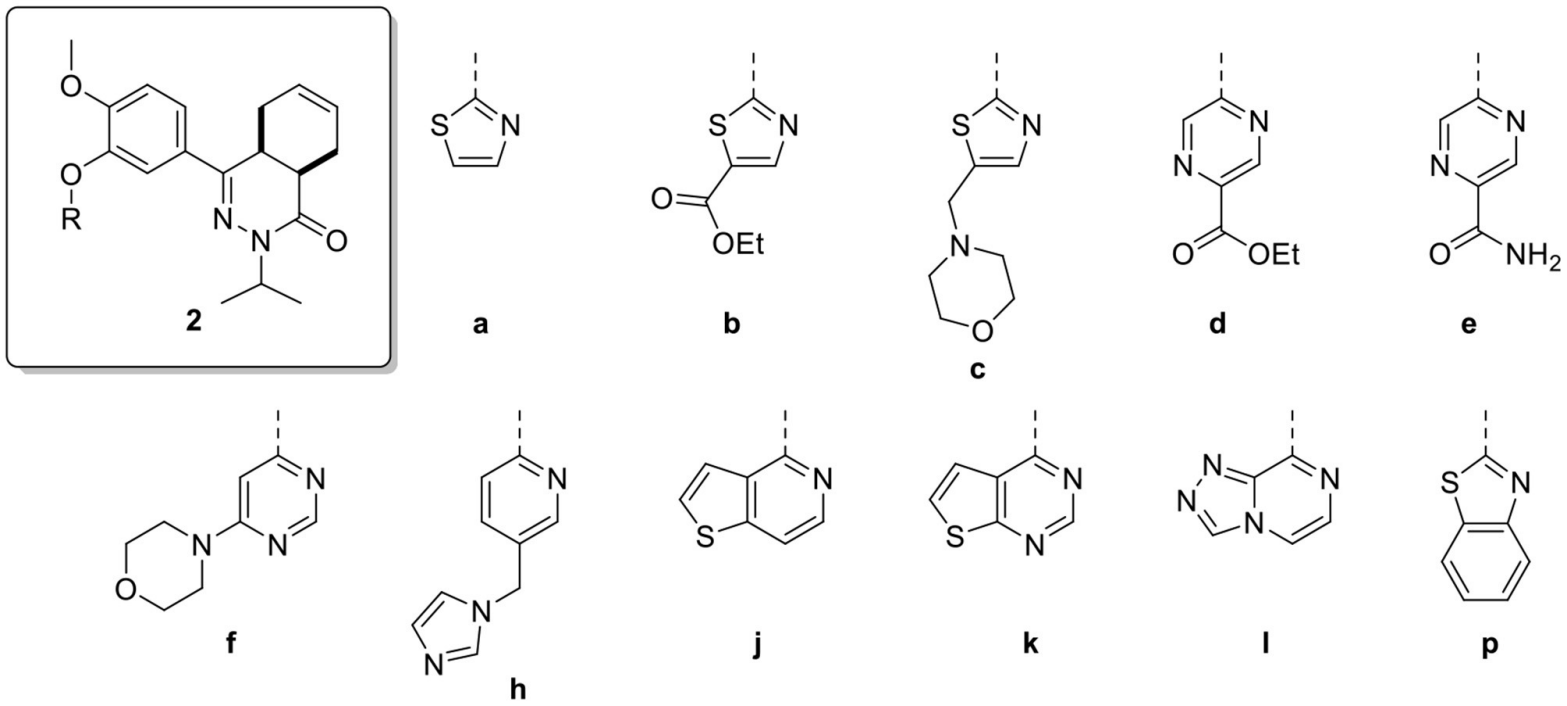					
**#**	**NPD-**	**TbrPDEB1 (% inh.)**	**#**	**NPD-**	**TbrPDEB1 (% inh.)**
**2a**	1162	12 ± 3	**2h**	1,164	49 ± 2
**2b**	1315	11 ± 0	**2j**	1,157	No inhibition
**2c**	1163	40 ± 4	**2k**	1,160	No inhibition
**2d**	1337	69 ± 1	**2l**	1,158	No inhibition
**2e**	3162	18 ± 9	**2p**	1,161	No inhibition
**2f**	1159	11 ± 8			

The computer-aided design of the pyridines on the different positions gave only a few active compounds (Table 2). With exception of **3m**, all substitutions on the 3- and 4-position of the pyridine ring resulted in no inhibition of TbrPDEB1 at 10 μM. A methyl ester substitution on the 5-position of the pyridine ring (**3c**) resulted in a moderate inhibition, while larger groups or a carboxylic acid were not tolerated on this position. The best results were obtained for substitutions on the 6-position next to the pyridine nitrogen (**3d**, **3h**, **3l**, **3o**, **3s**, and **3w**). With exception of the carboxylic acid functionality, all substitutions on this position resulted in a moderate inhibitory effect, suggesting that this is the ideal vector to fit the side groups into the active site. Although we observed relatively small differences between the different analogs, the best activities were obtained for *n*-butyl and furfuryl substituted diaryl ethers **3s** and **3w**, which both showed slightly more than 50% inhibition at 10 μM.

**Table 2:** Single point activities of structure guided diaryl ether phthalazinones against TbrPDEB1 at 10 μM.

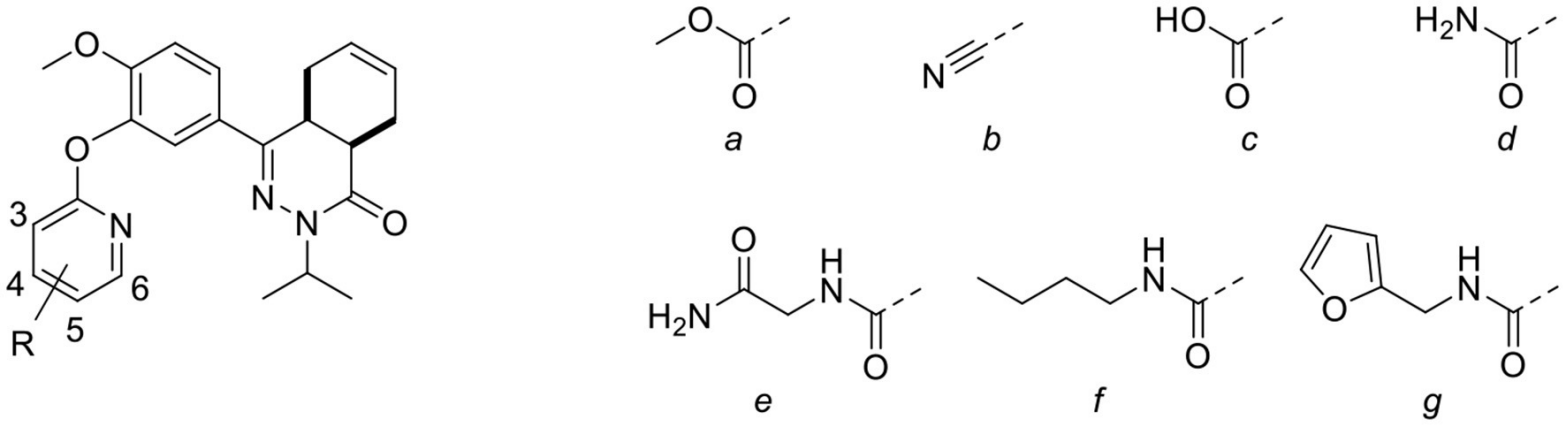									
**#**	**NPD**	**R**	**Pos**.	**TbrPDEB1 (% inh)**	**#**	**NPD**	**R**	**Pos**.	**TbrPDEB1 (% inh)**
**3a**	1338	a	3	No inhibition	**3m**	1,400	e	4	27 ± 0
**3b**	1340	b	4	No inhibition	**3n**	1,392	e	5	5 ± 1
**3c**	1339	a	5	50 ± 1	**3o**	1,397	e	6	40 ± 1
**3d**	1341	b	6	36 ± 6	**3p**	1,389	f	3	No inhibition
**3e**	1342	c	3	No inhibition	**3q**	3,167	f	4	No inhibition
**3f**	1394	c	4	No inhibition	**3r**	1,390	f	5	No inhibition
**3g**	1343	c	5	11 ± 6	**3s**	1,395	f	6	53 ± 3
**3h**	1393	c	6	No inhibition	**3t**	1,344	g	3	No inhibition
**3i**	1345	d	3	No inhibition	**3u**	1,444	g	4	No inhibition
**3j**	1398	d	4	No inhibition	**3v**	1,391	g	5	No inhibition
**3k**	3165	d	5	No inhibition	**3w**	1,396	g	6	51 ± 9
**3l**	1399	d	6	48 ± 10					

All compounds (**2d**, **2h**, **3c**, **3l**, **3s**, and **3w**) showing about 50% inhibition or higher at 10 μM in the single point assay were selected for a TbrPDEB1 full dose-response assay, their *in vitro* activity against *T. brucei* parasites and *in vitro* cytotoxicity for MRC-5 cells. In line with the results in the 10 μM assay, all selected diaryl ethers showed interesting inhibitory activity against TbrPDEB1 with pK_i_ values between 5.9 and 6.2 (Table 3). In the phenotypic assays, this set of compounds, except **3s**, showed an activity comparable to **1** with *T. brucei* IC_50_ values in the range of 7.9–25 μM. All compounds, with exception of **2h**, did not show cytotoxicity at the highest measured concentration (CC_50_ > 64 μM), resulting in an acceptable cytotoxicity profile for **2d**, **3c**, **3l**, and **3w**. These results suggest that the introduction of the ether functionality has no effect on the cellular activity when compared to **1**.

**Table 3:** *In vitro* activity of selected phthalazinones against TbrPDEB1, *T. brucei* parasites, and MRC-5 cells.

**#**	**TbrPDEB1 K_**i**_ (μM)**	***T. brucei* IC_**50**_ (μM)**	**MRC-5 CC_**50**_ (μM)**
**1**	0.1^a^	6.3^a^	35^a^
**2d**	0.6 ± 0.2	16 ± 10	>64
**2h**	1.3 ± 0.2	7.9 ± 8	31 ± 8
**3c**	0.8 ± 0.2	7.9 ± 8	>64
**3l**	0.8 ± 0.2	16 ± 10	>64
**3s**	1.3 ± 0.3	>64	>64
**3w**	1.0 ± 0.2	25 ± 20	>64

a *Reported by Blaazer et al. (2018)*.

## Conclusion

The computer-aided design of novel diaryl substituted tetrahydrophthalazinones resulted in the identification of several compounds with activities in the low micromolar range against TbrPDEB1 and devoid of cytotoxicity against MRC-5 cells. The results suggest a favorable position of modification for *para*-substituted 6-membered heteroaromatics (**2d**, **2h**, and **3c**) or 2,6-substituted pyridines (**3l**, **3s**, **3o**, and **3w**). The current set of compounds provides additional insight in the SAR for development of new selective TbrPDEB1 inhibitors. These results are important in the design of TbrPDEB1 selective inhibitors with adequate selectivity (>30-fold over human PDE4) and efficacy (IC_50_ < 1 μM) against this parasite.

## Data Availability Statement

The original contributions presented in the study are included in the article/Supplementary Material, further inquiries can be directed to the corresponding author.

## Author Contributions

EH, SK, JS, GS, and IE were involved in compound design, synthesis, and analysis. EH and AK were involved in virtual screening and docking. TM, PS, and MS were involved in the biochemical assays. DM, GC, and LM were involved in the phenotypic cellular assays. LM, GS, IE, and RL supervised the experiments and conceived the project. EH, GS, and RL integrated all data and wrote the manuscript. All authors contributed to the article and approved the submitted version.

## Conflict of Interest

The authors declare that the research was conducted in the absence of any commercial or financial relationships that could be construed as a potential conflict of interest.
