# Supplementary material for: Discovery of Diaryl Ether Substituted Tetrahydrophthalazinones as TbrPDEB1 Inhibitors Following Structure-Based Virtual Screening
Source: Front Chem. 2021 Jan 21;8:608030. doi: 10.3389/fchem.2020.608030 (PMC7859335; doi:10.3389/fchem.2020.608030)

Supplementary Material

Appendix A: Synthetic procedures

# Appendix B: Virtual screening hits after visual inspection sorted by class.

Appendix C. Docking pose of selected hits for synthesis.

1. **Appendix A: Synthetic procedures**
   1. 2-Methoxyphenyl methanesulfonate (**4**)

To an ice-cooled solution of 2-methoxyphenol (45 mL, 0.40 mol) and Et_3_N (95 mL, 0.69 mol) in DCM (120 mL) was added methanesulfonyl chloride (41 mL, 0.52 mol) in a dropwise fashion to give an orange suspension, which was stirred at rt for 1 h. The reaction mixture was quenched by adding water (350 mL). The aqueous phase was extracted with DCM (2 × 300 mL) and the organic phases were combined and washed with aq. Na_2_CO_3_ (1 M, 300 mL) and brine (300 mL). The organic phase was dried over MgSO_4_ and the solvent was removed *in vacuo* to yield the title compound (63 g, 78%) as an orange oil.

^1^H NMR (500 MHz, CDCl_3_): δ 7.35 – 7.23 (m, 2H), 7.06 – 6.93 (m, 2H), 3.89 (s, 3H), 3.17 (s, 3H). ^13^C NMR (126 MHz, CDCl_3_): δ 151.4, 138.3, 128.4, 124.5, 121.1, 112.9, 56.0, 38.2. LC-MS (ESI): *t*_R_ = 3.89 min, area: >98%, no ionization.

- 1. *(E)*-4-(4-Methoxy-3-((methylsulfonyl)oxy)phenyl)-4-oxobut-2-enoic acid (**5**)

To a yellow solution of **4** (24.4 g, 121 mmol) and furan-2,5-dione (14.2 g, 145 mmol) in DCM (200 mL) was slowly added AlCl_3_ (33.8 g, 253 mmol). The dark red solution was stirred at rt for 5 h. The dark red suspension was quenched by slowly pouring into ice-cooled aq. HCl (2 M, 400 mL). The organic phase was separated and the aqueous phase was extracted with DCM (200 mL) and EtOAc (2 × 200 mL). The organic phases were combined and concentrated *in vacuo* till crystallization was initiated. Further removal of solvents was stopped and the mixture was allowed further crystallization to yield the title compound (14.3 g, 40%) as yellow crystals.

^1^H NMR (500 MHz, DMSO-*d*_6_): δ 8.11 (dd, *J* = 8.8, 2.2 Hz, 1H), 7.97 – 7.87 (m, 2H), 7.37 (d, *J* = 8.8 Hz, 1H), 6.69 (d, *J* = 15.5 Hz, 1H), 3.96 (s, 3H), 3.43 (s, 3H). ^13^C NMR (126 MHz, DMSO-*d*_6_): δ 187.4, 166.8, 156.7, 138.3, 136.2, 133.3, 130.7, 129.6, 124.6, 114.0, 57.1, 38.9. LC-MS (ESI): *t*_R_ = 3.37 min, area: >98%, *m/z* 301 [M + H]^+^.

- 1. *trans*-6-(4-Methoxy-3-((methylsulfonyl)oxy)benzoyl)cyclohex-3-enecarboxylic acid (**6**)

A mixture of **5** (10.0 g, 33.3 mmol) in a solution of buta-1,3-diene in THF (2 M, 33.3 mL, 66.6 mmol) was divided over two microwave vials. The reaction mixtures were stirred at 140 °C (pressure increased to ~11 bar) for 1.5 h by microwave irradiation. The two reactions were pooled and concentrated *in vacuo* to give a slightly yellow powder. This solid was triturated with toluene and filtrated to yield the title compound (10.8 g, 92%) as a white solid.

^1^H NMR (500 MHz, DMSO-*d*_6_): δ 12.29 (s, 1H), 8.08 (dd, *J* = 8.8, 2.2 Hz, 1H), 7.87 (d, *J* = 2.1 Hz, 1H), 7.33 (d, *J* = 8.7 Hz, 1H), 5.81 – 5.67 (m, 2H), 3.95 (s, 3H), 3.78 (td, *J* = 11.2, 5.4 Hz, 1H), 3.41 (s, 3H), 2.81 (td, *J* = 11.3, 5.5 Hz, 1H), 2.49 – 2.40 (m, 1H), 2.40 – 2.28 (m, 1H), 2.23 – 2.10 (m, 1H), 1.94 – 1.80 (m, 1H). ^13^C NMR (126 MHz, DMSO-*d*_6_): δ 200.9, 176.4, 156.0, 138.2, 129.9, 129.7, 125.8, 125.7, 124.1, 113.8, 57.0, 42.3, 41.6, 38.9, 29.4, 28.4. LC-MS (ESI): *t*_R_ = 3.89 min, area: 97%, *m/z* 355 [M + H]^+^.

- 1. *cis-*4-(3-Hydroxy-4-methoxyphenyl)-2-isopropyl-4a,5,8,8a-tetrahydrophthalazin-1(2*H*)-one (**8**)

A suspension of **6** (3.5 g, 10 mmol), Cs_2_CO_3_ (6.5 g, 20 mmol) and isopropylhydrazine.HCl (3.3 g, 30 mmol) in EtOH (10 mL) were charged in a microwave vial. This suspension was stirred in a microwave at 100 °C for 6 h. The reaction mixture was slowly poured into an ice-cooled aq. HCl solution (1 M, 100 mL) upon which white solids formed. These white solids where filtered of and dried in vacuo to give a mixture of the mesylated (**7**) and demesylated products (**8**).

To a solution of the crude product (2.95 g, 7.52 mmol) in dry THF (20 mL) and MeOH (15 mL) was added aq. NaOH (1 M, 20 mL). The reaction mixture was stirred at 50 °C for 2 h. THF and MeOH were removed *in vacuo*. The aqueous mixture was slowly poured into an ice-cooled aq. HCl (1 M, 100 mL) upon which yellow-white solids appeared. The solids were removed by filtration and dried *in vacuo*. The product was purified using flash column chromatography (EtOAc/cyclohexane: 0-100%) to yield the title compound (1.50 g, 47% over 2 steps) as a white powder.

^1^H NMR (500 MHz, CDCl_3_): δ 7.52 (d, *J* = 2.2 Hz, 1H), 7.30 (dd, *J* = 8.4, 2.2 Hz, 1H), 6.88 (d, *J* = 8.5 Hz, 1H), 5.82 (s, 1H), 5.81 – 5.64 (m, 2H), 5.05 (hept, *J* = 6.6 Hz, 1H), 3.95 (s, 3H), 3.29 (dt, *J* = 11.6, 5.8 Hz, 1H), 3.06 – 2.96 (m, 1H), 2.74 (t, *J* = 6.0 Hz, 1H), 2.26 – 2.12 (m, 2H), 2.07 – 1.97 (m, 1H), 1.33 (d, *J* = 6.6 Hz, 3H), 1.21 (d, *J* = 6.7 Hz, 3H). ^13^C NMR (126 MHz, CDCl_3_): δ 166.5, 153.6, 148.0, 145.8, 128.7, 126.0, 124.0, 118.2, 111.9, 110.2, 56.0, 46.6, 34.7, 31.0, 23.2, 22.4, 20.6, 20.2. LC-MS (ESI): *t*_R_ = 4.38 min, area: >98%, *m/z* 315 [M + H]^+^. HRMS (ESI) *m/z*: [M + H]^+^ calcd. for C_18_H_23_N_2_O_3_ 315.1703, found 315.1689.

- 1. General procedure for nucleophilic aromatic substitution

To a suspension of **8** (1 equiv.) and Cs_2_CO_3_ (2.5 equiv.) in DMF (3 mL/equiv.) in a microwave vial was added the corresponding aromatic chloride (1 - 1.5 equiv.). This mixture was heated to 100 °C for 3 h under microwave irradiation, unless indicated otherwise. The reaction was quenched by slowly pouring it into ice-cooled aq. 1 M HCl solution and the product was extracted with EtOAc (3 ×). The organic phases were combined, washed twice with brine, dried over MgSO_4_ and concentrated *in vacuo*. The crude product was purified as indicated.

- 1. *cis*-2-Isopropyl-4-(4-methoxy-3-(thiazol-2-yloxy)phenyl)-4a,5,8,8a-tetrahydrophthalazin-1(2*H*)-one (**2a**)

This compound was synthesized according to the general procedure starting with **8** (0.15 g, 0.48 mmol) and 2-chlorothiazole (0.10 g, 0.81 mmol). The reaction mixture was stirred in a microwave at 100 °C for 11 h. The crude product was purified by column chromatography using EtOAc/n-heptane (gradient: 10-50%) to yield the title compound (110 mg, 58%) as a white solid.

^1^H NMR (500 MHz, CDCl_3_): δ 7.80 (s, 1H), 7.69 (dd, *J* = 8.7, 3.8 Hz, 1H), 7.17 (s, 1H), 7.05 (d, *J* = 8.7 Hz, 1H), 6.79 (d, *J* = 3.8 Hz, 1H), 5.81 – 5.61 (m, 2H), 5.02 (hept, *J* = 6.6 Hz, 1H), 3.89 – 3.80 (m, 3H), 3.27 (dt, *J* = 11.6, 5.8 Hz, 1H), 3.04 – 2.93 (m, 1H), 2.72 (t, *J* = 6.1 Hz, 1H), 2.24 – 2.10 (m, 2H), 2.07 – 1.96 (m, 1H), 1.30 (d, *J* = 6.6 Hz, 3H), 1.18 (d, *J* = 6.7 Hz, 3H). ^13^C NMR (126 MHz, CDCl_3_): δ 174.2, 166.4, 152.5, 152.3, 143.9, 137.4, 128.6, 126.0, 124.8, 123.9, 119.8, 112.8, 112.7, 56.2, 46.7, 34.7, 30.9, 23.1, 22.3, 20.6, 20.2. LC-MS (ESI): *t*_R_ = 4.97 min, area: >98%, *m/z* 398 [M + H]^+^. HRMS (ESI) *m/z*: [M + H]^+^ calcd. for C_21_H_24_N_3_O_3_S 398.1533, found 398.1525.

- 1. Ethyl 2-(5-(*cis*-3-isopropyl-4-oxo-3,4,4a,5,8,8a-hexahydrophthalazin-1-yl)-2-methoxyphenoxy)thiazole-5-carboxylate (**2b**)

This compound was synthesized according to the general procedure starting with **8** (400 mg, 1.27 mmol) and ethyl 2-chlorothiazole-5-carboxylate (293 mg, 1.53 mmol). The mixture was stirred 100 °C for 10 h. The crude product was purified by column chromatography using EtOAc/n-heptane (gradient: 10-50%) to obtain a white solid (250 mg, 35%).

^1^H NMR (500 MHz, CDCl_3_): δ 7.90 – 7.83 (m, 1H), 7.83 – 7.77 (m, 1H), 7.72 (d, *J* = 8.7 Hz, 1H), 7.11 – 7.02 (m, 1H), 5.82 – 5.61 (m, 2H), 5.11 – 4.94 (m, 1H), 4.39 – 4.22 (m, 2H), 3.86 (d, *J* = 1.8 Hz, 3H), 3.28 (dt, *J* = 11.6, 5.7 Hz, 1H), 3.07 – 2.92 (m, 1H), 2.74 (t, *J* = 6.0 Hz, 1H), 2.27 – 2.10 (m, 2H), 2.09 – 1.92 (m, 1H), 1.39 – 1.32 (m, 3H), 1.30 (d, *J* = 6.6 Hz, 3H), 1.19 (d, *J* = 6.7 Hz, 3H). ^13^C NMR (126 MHz, CDCl_3_): δ 177.7, 166.4, 161.3, 152.3, 152.0, 144.5, 143.4, 128.7, 126.0, 125.3, 123.8, 123.0, 119.7, 112.9, 61.4, 56.2, 46.7, 34.7, 31.0, 23.0, 22.3, 20.6, 20.2, 14.3. LC-MS (ESI): *t*_R_ = 5.37 min, area: 95%, *m/z* 470 [M + H]^+^. HRMS (ESI) *m/z*: [M + H]^+^ calcd. for C_24_H_28_N_3_O_5_S 470.1744, found 470.1750.

- 1. *cis*-2-Isopropyl-4-(4-methoxy-3-((5-(morpholinomethyl)thiazol-2-yl)oxy)phenyl)-4a,5,8,8a-tetrahydrophthalazin-1(2*H*)-one (**2c**)

This compound was synthesized according to the general procedure starting with **8** (150 mg, 0.477 mmol) and 4-((2-chlorothiazol-5-yl)methyl)morpholine (125 mg, 0.573 mmol). The crude product was purified by column chromatography using EtOAc/n-heptane (gradient: 10-50%) to yield the title compound (110 mg, 45%) as a white solid.

^1^H NMR (500 MHz, CDCl_3_): δ 7.79 (d, *J* = 2.2 Hz, 1H), 7.69 (dd, *J* = 8.6, 2.2 Hz, 1H), 7.05 (d, *J* = 8.7 Hz, 1H), 6.99 (s, 1H), 5.84 – 5.63 (m, 2H), 5.04 (hept, *J* = 6.5 Hz, 1H), 3.88 (s, 3H), 3.72 (t, *J* = 4.7 Hz, 4H), 3.59 (s, 2H), 3.27 (dt, *J* = 11.5, 5.7 Hz, 1H), 3.06 – 2.95 (m, 1H), 2.73 (t, *J* = 5.9 Hz, 1H), 2.50 (s, 4H), 2.25 – 2.12 (m, 2H), 2.08 – 1.96 (m, 1H), 1.31 (d, *J* = 6.6 Hz, 3H), 1.19 (d, *J* = 6.7 Hz, 3H). ^13^C NMR (126 MHz, CDCl_3_): δ 174.0, 166.4, 152.5, 152.3, 143.7, 128.6, 126.0, 124.7, 123.9, 119.8, 112.8, 66.8, 56.2, 55.4, 53.1, 46.7, 34.7, 31.0, 23.1, 22.3, 20.6, 20.2. LC-MS (ESI): *t*_R_ = 3.53 min, area: 97%, *m/z* 497 [M + H]^+^. HRMS (ESI) *m/z*: [M + H]^+^ calcd. for C_26_H_33_N_4_O_4_S 497.2217, found 497.2206.

- 1. Methyl 5-(5-(*cis*-3-isopropyl-4-oxo-3,4,4a,5,8,8a-hexahydrophthalazin-1-yl)-2-methoxyphenoxy)pyrazine-2-carboxylate (**2d**)

This compound was synthesized according to the general procedure starting with **8** (150 mg, 0.477 mmol) and methyl 5-chloro-pyrazine-2-carboxylate (99 mg, 0.57 mmol). The mixture was heated to 50 °C for 1 h. The crude product was purified by column chromatography using EtOAc/cyclohexane (gradient: 0-30%) to give the title compound as a white solid (150 mg, 70%).

^1^H NMR (500MHz, CDCl_3_): δ 8.81 (d, *J* = 1.2 Hz, 1H), 8.55 (d, *J* = 1.2 Hz, 1H), 7.74 (d, *J* = 2.1 Hz, 1H), 7.70 (dd, *J* = 8.6, 2.2 Hz, 1H), 7.05 (d, *J* = 8.7 Hz, 1H), 5.83 – 5.62 (m, 2H), 5.03 (hept, *J* = 6.5 Hz, 1H), 4.01 (s, 3H), 3.78 (s, 3H), 3.29 (dt, *J* = 11.6, 5.7 Hz, 1H), 3.04 – 2.97 (m, 1H), 2.73 (t, *J* = 5.9 Hz, 1H), 2.24 – 2.14 (m, 2H), 2.09 – 1.99 (m, 1H), 1.30 (d, *J* = 6.6 Hz, 3H), 1.18 (d, *J* = 6.7 Hz, 3H). ^13^C NMR (126 MHz, CDCl_3_): δ 166.5, 164.4, 161.4, 152.6, 152.4, 144.3, 141.3, 137.3, 135.0, 128.8, 126.1, 124.9, 124.0, 120.2, 112.4, 56.1, 53.0, 46.8, 34.8, 31.1, 23.2, 22.4, 20.7, 20.3. LC-MS (ESI): *t*_R_ = 4.74 min, area: 96%, *m/z* 451 [M + H]^+^. HRMS (ESI) *m/z*: [M + H]^+^ calcd. for C_24_H_27_N_4_O_5_ 451.1976, found 451.1979.

- 1. 5-(5-(*cis*-3-Isopropyl-4-oxo-3,4,4a,5,8,8a-hexahydrophthalazin-1-yl)-2-methoxyphenoxy)pyrazine-2-carboxamide (**2e**)

This compound was synthesized according to the general procedure starting with **8** (100 mg, 0.318 mmol) and 5- chloropyrazine-2-carboxamide (60.1 mg, 0.382 mmol). The mixture was stirred at 50 °C for 5 h. The crude product was purified by column chromatography using EtOAc/cyclohexane (gradient: 0-50%) to give the title compound as a white solid (52 mg, 38%).

^1^H NMR (500 MHz, CDCl_3_) δ 9.08 (s, 1H), 8.61 (s, 1H), 7.76 (d, *J* = 2.2 Hz, 1H), 7.71 (dd, *J* = 8.6, 2.2 Hz, 1H), 7.09 (d, 6.3 Hz, 1H), 7.05 (d, 6.3 Hz, 1H), 5.94 (d, *J* = 3.9 Hz, 1H), 5.85 – 5.75 (m, 1H), 5.74 – 5.64 (m, 1H), 5.05 (hept, *J* = 6.8 Hz, 1H), 3.80 (s, 3H), 3.38 – 3.25 (m, 1H), 3.09 – 2.95 (m, 1H), 2.77 (t, *J* = 5.9 Hz, 1H), 2.29 – 2.14 (m, 2H), 2.12 – 1.99 (m, 1H), 1.32 (d, *J* = 6.6 Hz, 3H), 1.21 (d, *J* = 6.7 Hz, 3H). ^13^C NMR (126 MHz, CDCl_3_) δ 166.8, 165.1, 157.7, 152.5, 152.4, 141.2, 140.4, 138.1, 137.6, 128.7, 126.1, 124.5, 123.8, 120.1, 112.2, 56.0, 46.8, 34.7, 31.0, 23.1, 22.3, 20.6, 20.2. LC-MS (ESI): *t*_R_ = 4.24 min, area: 96%, *m/z* 436 [M + H]^+^. HRMS (ESI) *m/z*: [M + H]^+^ calcd. for C_23_H_26_N_5_O_4_ 436.1979, found 436.1989.

- 1. *cis*-2-Isopropyl-4-(4-methoxy-3-((6-morpholinopyrimidin-4-yl)oxy)phenyl)-4a,5,8,8a-tetrahydrophthalazin-1(2*H*)-one (**2f**)

This compound was synthesized according to the general procedure starting with **8** (150 mg, 0.477 mmol) and 4-(6-chloropyrimidin-4-yl)morpholine (95 mg, 0.50 mmol). The mixture was stirred at 100 °C for 5 h. Residual DMF was removed by co-evaporation with toluene to yield the title compound (165 mg, 72%) as a white powder.

^1^H NMR (500 MHz, DMSO-*d*_6_): δ 8.14 (s, 1H), 7.72 (dd, *J* = 8.7, 2.2 Hz, 1H), 7.62 (d, *J* = 2.2 Hz, 1H), 7.19 (d, *J* = 8.7 Hz, 1H), 6.34 (s, 1H), 5.73 – 5.58 (m, 2H), 4.86 (hept, *J* = 6.6 Hz, 1H), 3.75 (s, 3H), 3.70 – 3.63 (m, 4H), 3.61 – 3.53 (m, 4H), 3.44 (dt, *J* = 11.5, 5.8 Hz, 1H), 2.80 (t, *J* = 6.0 Hz, 1H), 2.77 – 2.69 (m, 1H), 2.20 – 2.06 (m, 2H), 1.89 – 1.69 (m, 1H), 1.21 (d, *J* = 6.5 Hz, 3H), 1.11 (d, *J* = 6.7 Hz, 3H). ^13^C NMR (126 MHz, DMSO-*d*_6_): δ 170.0, 166.5, 164.4, 157.7, 153.5, 153.2, 141.8, 128.1, 126.3, 124.7, 124.5, 120.5, 113.2, 86.2, 66.2, 56.4, 46.1, 44.5, 34.3, 30.3, 23.0, 22.4, 20.9, 20.6. LC-MS (ESI): *t*_R_ = 4.69 min, area: 96%, *m/z* 478 [M + H]^+^. HRMS (ESI) *m/z*: [M + H]^+^ calcd. for C_26_H_32_N_5_O_4_ 478.2449, found 478.2454.

- 1. *cis*-4-(3-((5-((1*H*-Imidazol-1-yl)methyl)pyridin-2-yl)oxy)-4-methoxyphenyl)-2-isopropyl-4a,5,8,8a-tetrahydrophthalazin-1(2*H*)-one (**2h**)

This compound was synthesized according to the general procedure starting with **8** (150 mg, 0.477 mmol) and 5-((1*H*-imidazol-1-yl)methyl)-2-chloropyridine (111 mg, 0.573 mmol). The crude product was dissolved DMSO and subjected to reversed phase column chromatography using H_2_O/MeCN + 0.1% formic acid (gradient: 0-100%) to obtain the title compound (62 mg, 27%) as a colourless oil.

^1^H NMR (500 MHz, CDCl_3_): δ 8.04 (s, 1H), 7.75 (s, 1H), 7.71 – 7.62 (m, 2H), 7.52 (dd, *J* = 8.6, 2.5 Hz, 1H), 7.11 (s, 1H), 7.08 – 7.01 (m, 1H), 7.01 – 6.84 (m, 2H), 5.82 – 5.58 (m, 2H), 5.10 (s, 2H), 5.07 – 4.92 (m, 1H), 3.82 – 3.70 (m, 3H), 3.34 – 3.19 (m, 1H), 3.04 – 2.90 (m, 1H), 2.71 (s, 1H), 2.25 – 2.11 (m, 2H), 2.08 – 1.93 (m, 1H), 1.28 (d, *J* = 6.8, 3H), 1.17 (d, *J* = 6.8, 3H). ^13^C NMR (126 MHz, CDCl_3_) δ 166.4, 163.6, 152.9, 152.9, 146.6, 142.1, 138.9, 128.6, 128.5, 126.0, 125.6, 124.1, 124.0, 120.5, 119.2, 112.3, 111.2, 56.0, 48.1, 46.6, 34.7, 31.0, 23.1, 22.3, 20.6, 20.2. (One imidazole carbon not detected). LC-MS (ESI): *t*_R_ = 3.52 min, area: >98%, *m/z* 472 [M + H]^+^. HRMS (ESI) *m/z*: [M + H]^+^ calcd. for C_27_H_30_N_5_O_3_ 472.2343, found 472.2352.

- 1. *cis*-2-Isopropyl-4-(4-methoxy-3-(thieno[3,2-c]pyridin-4-yloxy)phenyl)-4a,5,8,8a-tetrahydrophthalazin-1(2*H*)-one (**2j**)

This compound was synthesized according to the general procedure starting with **8** (150 mg, 0.477 mmol) and 4-chlorothieno[3,2-c]pyridine (121 mg, 0.716 mmol). The mixture was stirred at rt for 2 h. The crude product was purified by column chromatography using EtOAc/n-heptane (gradient: 10-50%) to yield the title compound (110 mg, 50%) as a white solid.

^1^H NMR (500 MHz, CDCl_3_): δ 7.95 (d, *J* = 5.7 Hz, 1H), 7.77 (d, *J* = 2.2 Hz, 1H), 7.75 – 7.68 (m, 2H), 7.54 – 7.47 (m, 2H), 7.08 (d, *J* = 8.7 Hz, 1H), 5.82 – 5.63 (m, 2H), 5.04 (hept, *J* = 6.6 Hz, 1H), 3.80 (s, 3H), 3.31 (dt, *J* = 11.5, 5.7 Hz, 1H), 3.06 – 2.96 (m, 1H), 2.75 (t, *J* = 6.1 Hz, 1H), 2.26 – 2.15 (m, 2H), 2.11 – 1.99 (m, 1H), 1.31 (d, *J* = 6.5 Hz, 3H), 1.19 (d, *J* = 6.7 Hz, 3H). ^13^C NMR (126 MHz, CDCl_3_): δ 166.4, 158.2, 153.2, 153.0, 149.7, 142.3, 140.2, 128.5, 126.0, 124.3, 124.0, 121.1, 120.8, 112.6, 112.4, 56.1, 46.6, 34.7, 31.0, 23.1, 22.4, 20.6, 20.2. LC-MS (ESI): *t*_R_ = 5.43 min, area: 97%, *m/z* 448 [M + H]^+^. HRMS (ESI) *m/z*: [M + H]^+^ calcd. for C_25_H_26_N_3_O_3_S 448.1689, found 448.1689.

- 1. *cis*-2-Isopropyl-4-(4-methoxy-3-(thieno[2,3-d]pyrimidin-4-yloxy)phenyl)-4a,5,8,8a-tetrahydrophthalazin-1(2*H*)-one (**2k**)

This compound was synthesized according to the general procedure starting with **8** (150 mg, 0.477 mmol) and 4-chlorothieno[2,3-d]pyrimidine (122 mg, 0.716 mmol). The reaction mixture was stirred at rt for 1 h. The crude product was purified by column chromatography using EtOAc/n-heptane (gradient: 10-50%) to yield the title compound (50 mg, 23%) as a white solid.

^1^H NMR (500 MHz, DMSO-*d*_6_) δ 8.59 (s, 1H), 7.98 (d, *J* = 5.9 Hz, 1H), 7.86 – 7.79 (m, 2H), 7.69 (d, *J* = 5.9 Hz, 1H), 7.27 (d, *J* = 9.2 Hz, 1H), 5.73 – 5.58 (m, 2H), 4.86 (hept, *J* = 6.4 Hz, 1H), 3.74 (s, 3H), 3.45 (dt, *J* = 11.6, 5.8 Hz, 1H), 2.81 (t, *J* = 6.1 Hz, 1H), 2.78 – 2.68 (m, 1H), 2.21 – 2.09 (m, 2H), 1.88 – 1.76 (m, 1H), 1.21 (d, *J* = 6.6 Hz, 3H), 1.12 (d, *J* = 6.7 Hz, 3H). ^13^C NMR (126 MHz, DMSO-*d*_6_): δ 169.4, 166.5, 163.3, 153.5, 153.4, 152.8, 141.1, 128.4, 127.9, 126.3, 125.4, 124.5, 120.8, 119.0, 118.5, 113.4, 56.5, 46.2, 34.3, 30.3, 23.0, 22.4, 20.9, 20.5. LC-MS (ESI): *t*_R_ = 5.22 min, area: >98%, *m/z* 449 [M + H]^+^. HRMS (ESI) *m/z*: [M + H]^+^ calcd. for C_24_H_25_N_4_O_3_S 449.1642, found 449.1628.

- 1. *cis*-4-(3-([1,2,4]Triazolo[4,3-a]pyrazin-8-yloxy)-4-methoxyphenyl)-2-isopropyl-4a,5,8,8a-tetrahydrophthalazin-1(2*H*)-one (**2l**)

This compound was synthesized according to the general procedure starting with **8** (150 mg, 0.477 mmol) and 8-chloro-[1,2,4]triazolo[4,3-a]pyrazine (111 mg, 0.716 mmol). The reaction mixture was stirred at rt for 1 h. The reaction mixture was quenched by pouring into ice-cooled aq. HCl (1 M, 10 mL). The solids were filtered off and taken up in EtOAc (30 mL), which was washed with brine (2 x 30 mL), dried over MgSO_4_ and concentrated *in vacuo* to give the title compound (170 mg, 82%) as a white solid.

^1^H NMR (500 MHz, DMSO-*d*_6_): δ 9.48 (s, 1H), 8.32 (d, *J* = 4.7 Hz, 1H), 7.85 (d, *J* = 2.2 Hz, 1H), 7.82 (dd, *J* = 8.6, 2.3 Hz, 1H), 7.34 (d, *J* = 4.7 Hz, 1H), 7.28 (d, *J* = 8.7 Hz, 1H), 5.76 – 5.57 (m, 2H), 4.87 (hept, *J* = 6.7 Hz, 1H), 3.77 (s, 3H), 3.45 (dt, *J* = 11.5, 5.8 Hz, 1H), 2.82 (s, 1H), 2.79 – 2.68 (m, 1H), 2.31 – 2.01 (m, 2H), 1.92 – 1.75 (m, 1H), 1.21 (d, *J* = 6.6 Hz, 3H), 1.12 (d, *J* = 6.8 Hz, 3H). ^13^C NMR (126 MHz, DMSO-*d*_6_): δ 166.5, 153.4, 152.8, 152.4, 140.9, 138.8, 128.4, 126.8, 126.3, 125.4, 124.5, 120.8, 115.1, 113.5, 100.0, 56.5, 46.2, 34.3, 30.3, 23.0, 22.4, 20.9, 20.5. LC-MS (ESI): *t*_R_ = 4.08 min, area: >98%%, *m/z* 433 [M + H]^+^. HRMS (ESI) *m/z*: [M + H]^+^ calcd. for C_23_H_25_N_6_O_3_ 433.1983, found 433.1974.

- 1. *cis*-4-(3-(Benzo[d]thiazol-2-yloxy)-4-methoxyphenyl)-2-isopropyl-4a,5,8,8a-tetrahydrophthalazin-1(2*H*)-one (**2p**)

This compound was synthesized according to the general procedure starting with **8** (150 mg, 0.477 mmol) and 2-chlorobenzo[d]thiazole (81 mg, 0.48 mmol). In contrast to the general procedure, this reaction mixture was stirred at rt overnight. The title compound (132 mg, 61%) was obtained after work-up as a white powder.

^1^H NMR (500 MHz, DMSO-*d*_6_): δ 7.96 (d, *J* = 2.2 Hz, 1H), 7.92 (d, *J* = 8.0 Hz, 1H), 7.87 (dd, *J* = 8.7, 2.2 Hz, 1H), 7.66 (d, *J* = 8.0 Hz, 1H), 7.41 (t, *J* = 7.7 Hz, 1H), 7.36 – 7.27 (m, 2H), 5.74 – 5.57 (m, 2H), 4.87 (hept, *J* = 6.7 Hz, 1H), 3.83 (s, 3H), 3.48 (dt, *J* = 11.6, 5.7 Hz, 1H), 2.83 (s, 1H), 2.79 – 2.68 (m, 1H), 2.20 – 2.08 (m, 2H), 1.92 – 1.75 (m, 1H), 1.22 (d, *J* = 6.6 Hz, 3H), 1.13 (d, *J* = 6.7 Hz, 3H). ^13^C NMR (126 MHz, DMSO-*d*_6_): δ 172.5, 166.5, 153.2, 152.4, 149.0, 143.3, 132.3, 128.5, 126.9, 126.3, 126.1, 124.5, 124.5, 122.7, 121.5, 120.4, 114.1, 56.7, 46.2, 34.2, 30.3, 23.0, 22.4, 20.9, 20.5. LC-MS (ESI): *t*_R_ = 5.60 min, area: 98%, *m/z* 448 [M + H]^+^. HRMS (ESI) *m/z*: [M + H]^+^ calcd. for C_25_H_26_N_3_O_3_S 448.1689, found 448.1677.

- 1. Methyl 2-(5-(*cis*-3-isopropyl-4-oxo-3,4,4a,5,8,8a-hexahydrophthalazin-1-yl)-2-methoxyphenoxy)nicotinate (**3a**)

This compound was synthesized according to the general procedure starting with **8** (1.20 g, 3.82 mmol) and methyl 2-chloronicotinate (0.74 mL, 5.7 mmol). The crude product was purified by column chromatography using EtOAc/cyclohexane (gradient: 30-55% + 1% AcOH) to give the title compound as a white solid (876 mg, 51%).

^1^H NMR (500 MHz, DMSO-*d*_6_): δ 8.28 (dd, *J* = 7.6, 1.9 Hz, 1H), 8.24 (dd, *J* = 4.8, 1.9 Hz, 1H), 7.73 (dd, *J* = 8.7, 2.1 Hz, 1H), 7.63 (d, *J* = 2.1 Hz, 1H), 7.24 – 7.17 (m, 2H), 5.72 – 5.59 (m, 2H), 4.86 (hept, *J* = 6.3 Hz, 1H), 3.87 (s, 3H), 3.70 (s, 3H), 3.45 (dt, *J* = 11.5, 5.7 Hz, 1H), 2.80 (t, *J* = 5.7 Hz, 1H), 2.77 – 2.68 (m, 1H), 2.19 – 2.07 (m, 2H), 1.87 – 1.77 (m, 1H), 1.21 (d, *J* = 6.5 Hz, 3H), 1.12 (d, *J* = 6.7 Hz, 3H). ^13^C NMR (126 MHz, DMSO-*d*_6_): δ 166.1, 164.6, 160.7, 153.2, 152.7, 151.0, 141.9, 141.7, 127.9, 125.9, 124.2, 124.1, 120.1, 118.6, 113.6, 112.9, 56.0, 52.5, 45.7, 33.9, 29.9, 22.6, 22.0, 20.5, 20.1. LC-MS (ESI): *t*_R_ = 5.13 min, area: >98%, *m/z* 450 [M + H]^+^. HRMS (ESI) *m/z*: [M + H]^+^ calcd. for C_25_H_28_N_3_O_5_ 450.2023, found 450.2023.

- 1. 2-(5-(c*is*-3-Isopropyl-4-oxo-3,4,4a,5,8,8a-hexahydrophthalazin-1-yl)-2-methoxyphenoxy)isonicotinonitrile (**3b**)

This compound was synthesized according to the general procedure starting with **8** (1.1 g, 3.4 mmol) and 2-chloroisonicotinonitrile (712 mg, 5.14 mmol). The crude product was purified by column chromatography using EtOAc/cyclohexane (gradient: 0-20% + 2% AcOH) to give the title compound as a white solid (620 mg, 44%).

^1^H NMR (500 MHz, CDCl_3_): δ 8.28 (d, *J* = 5.0 Hz, 1H), 7.71 – 7.66 (m, 2H), 7.21 – 7.17 (m, 2H), 7.04 (d, *J* = 8.5 Hz, 1H), 5.72 (dd, *J* = 54.2, 9.3 Hz, 2H), 5.03 (hept, *J* = 6.3 Hz, 1H), 3.79 (s, 3H), 3.28 (dt, *J* = 11.5, 5.6 Hz, 1H), 3.04 – 2.96 (m, 1H), 2.73 (t, *J* = 5.8 Hz, 1H), 2.23 – 2.13 (m, 2H), 2.08 – 1.99 (m, 1H), 1.29 (d, *J* = 6.6 Hz, 3H), 1.18 (d, *J* = 6.7 Hz, 3H). ^13^C NMR (126 MHz, DMSO-*d*_6_): δ 166.1, 163.0, 153.0, 152.6, 148.9, 141.3, 128.0, 126.0, 124.5, 124.1, 122.5, 120.1, 120.1, 116.5, 113.6, 113.0, 56.0, 45.8, 33.9, 29.9, 22.6, 22.0, 20.5, 20.1. LC-MS (ESI): *t*_R_ = 5.01 min, area: >98%, *m/z* 417 [M + H]^+^. HRMS (ESI) *m/z*: [M + H]^+^ calcd. for C_24_H_25_N_4_O_3_ 417.1921, found 417.1915.

- 1. Methyl 6-(5-(*cis*-3-isopropyl-4-oxo-3,4,4a,5,8,8a-hexahydrophthalazin-1-yl)-2-methoxyphenoxy)nicotinate (**3c**)

This compound was synthesized according to the general procedure starting with **8** (1.20 g, 3.82 mmol) and methyl 6-chloronicotinate (0.98 g, 5.7 mmol). The crude product was purified by column chromatography using EtOAc/cyclohexane (gradient: 30-55% + 1% AcOH) to give the title compound as a white solid (1.06 g, 62%).

^1^H NMR (500 MHz, DMSO-*d*_6_): δ 8.64 (d, *J* = 2.4 Hz, 1H), 8.29 (dd, *J* = 8.7, 2.3 Hz, 1H), 7.76 (dd, *J* = 8.7, 2.0 Hz, 1H), 7.71 (d, *J* = 2.0 Hz, 1H), 7.23 (d, *J* = 8.7 Hz, 1H), 7.15 (d, *J* = 8.7 Hz, 1H), 5.71 – 5.59 (m, 2H), 4.86 (hept, *J* = 6.5 Hz, 1H), 3.84 (s, 3H), 3.72 (s, 3H), 3.44 (dt, *J* = 11.4, 5.7 Hz, 1H), 2.80 (t, *J* = 6.0 Hz, 1H), 2.76 – 2.69 (m, 1H), 2.19 – 2.09 (m, 2H), 1.86 – 1.76 (m, 1H), 1.21 (d, *J* = 6.5 Hz, 3H), 1.11 (d, *J* = 6.7 Hz, 3H).  ^13^C NMR (126 MHz, CDCl_3_) δ 166.1, 165.7, 164.9, 153.1, 152.6, 149.4, 141.5, 140.8, 128.0, 125.9, 124.6, 124.1, 120.9, 120.1, 113.1, 110.5, 56.0, 52.3, 45.8, 33.9, 29.9, 22.6, 22.0, 20.5, 20.1. LC-MS (ESI): *t*_R_ = 5.12 min, area: >98%, *m/z* 450 [M + H]^+^. HRMS (ESI) *m/z*: [M + H]^+^ calcd. for C_25_H_28_N_3_O_5_ 450.2023, found 450.2021.

- 1. 6-(5-(c*is*-3-Isopropyl-4-oxo-3,4,4a,5,8,8a-hexahydrophthalazin-1-yl)-2-methoxyphenoxy)picolinonitrile (**3d**)

This compound was synthesized according to the general procedure starting with **8** (1.2 g, 3.8 mmol) and 6-chloropicolinonitrile (793 mg, 5.73 mmol). The crude product was purified by column chromatography using EtOAc/cyclohexane (gradient: 0-20% + 2% TEA) to give the title compound (900 mg, 54%) as a white solid.

^1^H NMR (500 MHz, DMSO-*d_6_*): δ 8.07 (dd, *J* = 8.5, 7.3 Hz, 1H), 7.79 (dd, *J* = 8.7, 2.2 Hz, 1H), 7.76 (d, *J* = 7.3 Hz, 1H), 7.73 (d, *J* = 2.2 Hz, 1H), 7.42 (d, *J* = 8.4 Hz, 1H), 7.26 (d, *J* = 8.7 Hz, 1H), 5.72 – 5.58 (m, 2H), 4.87 (hept, *J* = 6.5 Hz, 1H), 3.75 (s, 3H), 3.45 (dt, *J* = 11.6, 5.8 Hz, 1H), 2.82 (t, *J* = 5.9 Hz, 1H), 2.78 – 2.68 (m, 1H), 2.13 (s, 2H), 1.87 – 1.78 (m, 1H), 1.22 (d, *J* = 6.6 Hz, 3H), 1.13 (d, *J* = 6.7 Hz, 3H). ^13^C NMR (126 MHz, DMSO-*d*_6_): δ 166.1, 163.0, 153.0, 152.5, 141.7, 141.1, 129.3, 128.0, 125.9, 124.7, 124.5, 124.1, 120.0, 117.1, 116.2, 113.2, 56.1, 45.8, 33.8, 29.9, 22.6, 22.0, 20.5, 20.1. LC-MS (ESI): *t*_R_ = 5.00 min, area: 96%, *m/z* 417 [M + H]^+^. HRMS (ESI) *m/z*: [M + H]^+^ calcd. for C_24_H_25_N_4_O_3_ 417.1921, found 417.1913.

- 1. 2-(5-(*cis*-3-Isopropyl-4-oxo-3,4,4a,5,8,8a-hexahydrophthalazin-1-yl)-2-methoxyphenoxy)nicotinic acid (**3e**)

To a solution of **3a** (875 mg, 1.95 mmol) in MeOH (20 mL) was added aq. NaOH (1.0 M, 10 mL, 10 mmol) and the reaction mixture was stirred at rt for 16 h. The reaction mixture was acidified with aq. 1 M HCl to pH 3 and was extracted with DCM (3 × 25 mL). The combined organic phases were dried over Na_2_SO_4_ and concentrated under reduced pressure to obtain the title compound as a white crystalline solid (690 mg, 81%)

^1^H NMR (500 MHz, DMSO-*d*_6_): δ 13.27 (s, 1H), 8.24 (dd, *J* = 7.5, 1.9 Hz, 1H), 8.19 (dd, *J* = 4.8, 1.9 Hz, 1H), 7.72 (dd, *J* = 8.7, 2.2 Hz, 1H), 7.63 (d, *J* = 2.2 Hz, 1H), 7.21 – 7.15 (m, 2H), 5.71 – 5.59 (m, 2H), 4.86 (hept, *J* = 6.6 Hz, 1H), 3.71 (s, 3H), 3.49 – 3.41 (m, 1H), 2.80 (t, *J* = 5.9 Hz, 1H), 2.77 – 2.69 (m, 1H), 2.19 – 2.07 (m, 2H), 1.86 – 1.77 (m, 1H), 1.21 (d, *J* = 6.6 Hz, 3H), 1.12 (d, *J* = 6.7 Hz, 3H). ^13^C NMR (126 MHz, CDCl_3_): δ 166.1, 165.8, 160.8, 153.2, 152.7, 150.4, 142.1, 141.6, 127.9, 125.9, 124.1, 124.1, 120.2, 118.5, 114.9, 112.8, 55.9, 45.8, 33.9, 29.9, 22.6, 22.0, 20.5, 20.1. LC-MS (ESI): *t*_R_ = 4.30 min, area: >98%, *m/z* 436 [M + H]^+^. HRMS (ESI) *m/z*: [M + H]^+^ calcd. for C_24_H_26_N_3_O_5_ 436.1867, found 436.1864.

- 1. 2-(5-(*cis*-3-Isopropyl-4-oxo-3,4,4a,5,8,8a-hexahydrophthalazin-1-yl)-2-methoxyphenoxy)isonicotinic acid (**3f**)

To a solution of **3b** (454 mg, 1.09 mmol) in 1,4-dioxane (14 mL) was added aq. NaOH (1.0 M, 14 mL, 14 mmol) and the reaction mixture was heated to 80 °C for 16 h. The reaction mixture was acidified with aq. HCl (1 M, 15 mL) and extracted with DCM (3 × 50 mL). The combined organic phases were dried over Na_2_SO_4_ and concentrated under reduced pressure to give the title compound as a white powder (487 mg, 99%)

^1^H NMR (500 MHz, CDCl_3_): δ 8.31 (d, *J* = 4.9 Hz, 1H), 7.72 (s, 1H), 7.68 (d, *J* = 8.6 Hz, 1H), 7.57 (d, *J* = 5.1 Hz, 2H), 7.04 (d, *J* = 8.6 Hz, 1H), 5.81 – 5.63 (m, 2H), 5.03 (hept, *J* = 6.7 Hz, 1H), 3.79 (s, 3H), 3.29 (dt, *J* = 11.4, 5.6 Hz, 1H), 3.05 – 2.97 (m, 1H), 2.79 – 2.69 (m, 1H), 2.24 – 2.13 (m, 2H), 2.10 – 1.98 (m, 1H), 1.30 (d, *J* = 6.5 Hz, 3H), 1.18 (d, *J* = 6.7 Hz, 3H). ^13^C NMR (126 MHz, CDCl_3_): δ 168.3, 166.7, 164.3, 153.1, 153.0, 148.5, 142.3, 140.4, 128.7, 126.1, 124.3, 124.1, 120.6, 117.8, 112.5, 111.4, 56.2, 46.9, 34.9, 31.1, 23.2, 22.5, 20.7, 20.3. LC-MS (ESI): *t*_R_ = 4.50 min, area: >98%, *m/z* 436 [M + H]^+^. HRMS (ESI) *m/z*: [M + H]^+^ calcd. for C_24_H_26_N_3_O_5_ 436.1867, found 436.1847.

- 1. 6-(5-(*cis*-3-Isopropyl-4-oxo-3,4,4a,5,8,8a-hexahydrophthalazin-1-yl)-2-methoxyphenoxy)nicotinic acid (**3g**)

To a solution of **3c** (1.1 g, 2.5 mmol) in MeOH (20 mL) was added aq. NaOH (1 M, 10 mL, 10 mmol) was added and the reaction mixture was stirred for 16 h. The reaction was quenched with aqueous 1 M HCl to pH 3 and was extracted with DCM (3 × 25 mL). The combined organic phases were dried over Na_2_SO_4_ and concentrated under reduced pressure. The crude product was purified by column chromatography using EtOAc/PhMe/cyclohexane + 1% AcOH (ratio: 0:1:1 to 1:1:1) to obtain the title compound as a white solid (791 mg, 74%).

^1^H NMR (500 MHz, CDCl_3_): δ 8.85 (d, *J* = 1.9 Hz, 1H), 8.33 (dd, *J* = 8.7, 2.2 Hz, 1H), 7.71 (d, *J* = 1.7 Hz, 1H), 7.69 (d, *J* = 8.6 Hz, 1H), 7.05 (d, *J* = 8.6 Hz, 2H), 5.81 – 5.63 (m, 2H), 5.03 (hept, *J* = 6.6 Hz, 1H), 3.79 (s, 3H), 3.29 (dt, *J* = 11.5, 5.7 Hz, 1H), 3.07 – 2.97 (m, 1H), 2.79 – 2.69 (m, 1H), 2.24 – 2.14 (m, 2H), 2.08 – 1.99 (m, 1H), 1.30 (d, *J* = 6.5 Hz, 3H), 1.18 (d, *J* = 6.7 Hz, 3H). ^13^C NMR (126 MHz, CDCl_3_): δ 169.8, 166.7, 166.6, 153.0, 152.9, 151.2, 142.1, 141.2, 128.7, 126.1, 124.5, 124.1, 120.5, 120.4, 112.5, 110.6, 56.2, 46.9, 34.9, 31.1, 23.2, 22.5, 20.7, 20.3. LC-MS (ESI): *t*_R_ = 4.48 min, area: >98%, *m/z* 436 [M + H]^+^. HRMS (ESI) *m/z*: [M + H]^+^ calcd. for C_24_H_26_N_3_O_5_ 436.1867, found 436.1864.

- 1. 6-(5-(*cis*-3-Isopropyl-4-oxo-3,4,4a,5,8,8a-hexahydrophthalazin-1-yl)-2-methoxyphenoxy)picolinic acid (**3h**)

To a solution of **3d** (600 mg, 1.44 mmol) in MeOH (20 mL) was added aq. NaOH (1 M, 10 mmol, 10 mL) and the reaction mixture was stirred at rt for 16 h. The reaction was quenched with aq. HCl (1 M, 10 mL) and the resulting mixture was extracted with DCM (3 × 50 mL). The combined organic phases were dried over Na_2_SO_4_ and concentrated under reduced pressure to give the title compound as an off-white solid (550 mg, 88%).  ^1^H NMR (300 MHz, CDCl_3_): δ 10.13 (s, 1H), 8.03 – 7.88 (m, 2H), 7.75 – 7.63 (m, 2H), 7.24 (d, *J* = 1.8 Hz, 1H), 7.05 (d, *J* = 8.4 Hz, 1H), 5.94 – 5.49 (m, 2H), 5.04 (hept, *J* = 6.4 Hz, 1H), 3.79 (s, 3H), 3.30 (dt, *J* = 11.4, 5.7 Hz, 1H), 3.07 – 2.94 (m, 1H), 2.75 – 2.67 (m, 1H), 2.29 – 2.12 (m, 2H), 2.11 – 1.98 (m, 1H), 1.31 (d, *J* = 6.6 Hz, 3H), 1.20 (d, *J* = 6.7 Hz, 3H). ^13^C NMR (126 MHz, CDCl_3_): δ 166.5, 163.6, 162.0, 152.8, 152.7, 143.7, 141.7, 141.6, 128.9, 126.2, 124.6, 124.0, 120.4, 118.5, 115.9, 112.4, 56.2, 46.9, 34.8, 31.2, 23.2, 22.4, 20.8, 20.3. LC-MS (ESI): *t*_R_ = 4.63 min, area: 96%, *m/z* 436 [M + H]^+^. HRMS (ESI) *m/z*: [M + H]^+^ calcd. for C_24_H_26_N_3_O_5_ 436.1867, found 436.1849.

- 1. 2-(5-(*cis*-3-Isopropyl-4-oxo-3,4,4a,5,8,8a-hexahydrophthalazin-1-yl)-2-methoxyphenoxy)nicotinamide (**3i**)

To a solution of **3e** (150 mg, 0.344 mmol) in DCM (3 mL) was added EDC∙HCl (198 mg, 1.03 mmol), HOBt∙H_2_O (158 mg, 1.03 mmol) and a solution of NH_3_ in MeOH (7 M, 0.98 mL, 6.9 mmol). The reaction mixture was stirred at rt for 72 h. The precipitate was filtered off and the resulting solution was concentrated under reduced pressure. The product was purified by column chromatography using EtOAc/cyclohexane (gradient: 20-50%) to obtain the title compound as a white solid (86 mg, 58%)

^1^H NMR (500 MHz, CDCl_3_): δ 8.59 (dd, *J* = 7.5, 1.6 Hz, 1H), 8.21 (dd, *J* = 4.7, 1.7 Hz, 1H), 7.86 – 7.76 (m, 2H), 7.70 (dd, *J* = 8.6, 1.9 Hz, 1H), 7.15 (dd, *J* = 7.5, 4.8 Hz, 1H), 7.04 (d, *J* = 8.7 Hz, 1H), 5.97 (s, 1H), 5.82 – 5.64 (m, 2H), 5.04 (hept, *J* = 6.4 Hz, 1H), 3.78 (s, 3H), 3.30 (dt, *J* = 11.5, 5.7 Hz, 1H), 3,15 – 2.89 (m, 1H), 2.81 – 2.71 (m, 1H), 2.24 – 2.15 (m, 2H), 2.10 – 1.99 (m, 1H), 1.31 (d, *J* = 6.5 Hz, 3H), 1.19 (d, *J* = 6.7 Hz, 3H). ^13^C NMR (126 MHz, DMSO-*d*_6_): δ 166.1, 165.2, 159.5, 153.2, 152.7, 149.1, 141.6, 140.7, 127.8, 125.9, 124.2, 124.1, 120.9, 118.9, 117.9, 112.8, 56.0, 45.8, 33.9, 30.0, 22.6, 22.0, 20.5, 20.1. LC-MS (ESI): *t*_R_ = 4.17 min, area: 98%, *m/z* 435 [M + H]^+^. HRMS (ESI) *m/z*: [M + H]^+^ calcd. for C_24_H_27_N_4_O_4_ 435.2027, found 435.2031.

- 1. 2-(5-(*cis*-3-Isopropyl-4-oxo-3,4,4a,5,8,8a-hexahydrophthalazin-1-yl)-2-methoxyphenoxy)isonicotinamide (**3j**)

To an ice-cooled solution of **3b** (100 mg, 0.240 mmol) in DMSO (1.2 mL) was added K_2_CO_3_ (133 mg, 0.96 mmol) and aq. H_2_O_2_ (30%, 0.25 mL) and the reaction mixture was stirred in an ice bath for 10 minutes. The reaction was diluted with ice-cold water (4 mL) and the resulting solids were filtered off and dried in a vacuum oven to obtain a sticky oil. The oil was dissolved in DCM/MeOH (ratio: 1:1) and concentrated to give an off-white solid, which was washed with water, dissolved in MeOH and concentrated to give the title compound as a white solid (103 mg, 99%).

^1^H NMR (500 MHz, CDCl_3_): δ 8.26 (d, *J* = 5.4 Hz, 1H), 7.72 (d, *J* = 2.2 Hz, 1H), 7.68 (dd, *J* = 8.6, 2.3 Hz, 1H), 7.39 – 7.30 (m, 2H), 7.04 (d, *J* = 8.6 Hz, 1H), 6.38 (s, 1H), 6.09 (s, 1H), 5.85 – 5.61 (m, 2H), 5.03 (hept, *J* = 6.6 Hz, 1H), 3.80 (s, 3H), 3.29 (dt, *J* = 11.6, 5.7 Hz, 1H), 3.06 – 2.94 (m, 1H), 2.78 – 2.66 (m, 1H), 2.25 – 2.12 (m, 2H), 2.10 – 1.95 (m, 1H), 1.30 (d, *J* = 6.6 Hz, 3H), 1.19 (d, *J* = 6.7 Hz, 3H). ^13^C NMR (126 MHz, CDCl_3_): δ 167.1, 166.6, 164.3, 153.0, 153.0, 148.6, 144.4, 142.3, 128.8, 126.1, 124.2, 124.1, 120.6, 115.9, 112.5, 108.9, 56.2, 46.8, 34.9, 31.1, 23.2, 22.5, 20.7, 20.3. LC-MS (ESI): *t*_R_ = 4.13 min, area: 98%, *m/z* 435 [M + H]^+^. HRMS (ESI) *m/z*: [M + H]^+^ calcd. for C_24_H_27_N_4_O_4_ 435.2027, found 435.2015.

- 1. 6-(5-(*cis*-3-Isopropyl-4-oxo-3,4,4a,5,8,8a-hexahydrophthalazin-1-yl)-2-methoxyphenoxy)nicotinamide (**3k**)

This compound was synthesized according to the general procedure starting with **8** (100 mg, 0.318 mmol) and 6-chloronicotinamide (59.8 mg, 0.382 mmol). The crude product was purified by column chromatography using EtOAc/cyclohexane (gradient: 0-50%) to give the title compound (34 mg, 24%) as a white solid.

^1^H NMR (500 MHz, CDCl_3_) δ 8.54 (d, *J* = 2.5 Hz, 1H), 8.24 (dd, *J* = 8.6, 2.5 Hz, 1H), 8.03 (s, 1H), 7.75 (dd, *J* = 8.6, 2.1 Hz, 1H), 7.68 (d, *J* = 2.3 Hz, 1H), 7.47 (s, 1H), 7.22 (d, *J* = 8.7 Hz, 1H), 7.09 (d, *J* = 8.6 Hz, 1H), 5.75 – 5.57 (m, 2H), 4.86 (hept, *J* = 6.7 Hz, 1H), 3.73 (s, 3H), 3.50 – 3.41 (m, 1H), 2.91 – 2.75 (m, 1H), 2.77 – 2.67 (m, 1H), 2.21 – 2.07 (m, 2H), 1.88 – 1.75 (m, 1H), 1.21 (d, *J* = 6.6 Hz, 3H), 1.12 (d, *J* = 6.8 Hz, 3H). ^13^C NMR (126 MHz, CDCl_3_) δ 171.2, 171.1, 169.7, 158.3, 157.9, 152.5, 146.9, 144.5, 133.1, 131.0, 130.1, 129.5, 129.2, 125.4, 118.1, 115.0, 61.1, 50.9, 39.0, 35.1, 27.8, 27.1, 25.6, 25.3. LC-MS (ESI): *t*_R_ = 4.17 min, area: 96%, *m/z* 435 [M + H]^+^. HRMS (ESI) *m/z*: [M + H]^+^ calcd. for C_24_H_27_N_4_O_4_ 435.2027, found 435.2028.

- 1. 6-(5-(*cis*-3-Isopropyl-4-oxo-3,4,4a,5,8,8a-hexahydrophthalazin-1-yl)-2-methoxyphenoxy)picolinamide (**3l**)

To an ice-cooled solution of **3d** (215 mg, 0.516 mmol) in DMSO (2.5 mL) was added K_2_CO_3_ (285 mg, 2.07 mmol) and aq. H_2_O_2_ (30%, 0.54 mL). The reaction mixture was stirred in an ice bath for 10 minutes. Ice-cold water (5 mL) was added and the resulting solids were filtered off, washed with water and dried in a vacuum oven to obtain the pure title compound as a white powder (160 mg, 71%).

^1^H NMR (300 MHz, CDCl_3_): δ 7.93 – 7.81 (m, 2H), 7.73 – 7.64 (m, 2H), 7.26 (s, 1H), 7.09 (d, *J* = 7.4 Hz, 1H), 7.03 (d, *J* = 8.4 Hz, 1H), 5.84 – 5.57 (m, 3H), 5.03 (hept, *J* = 6.3, 1H), 3.78 (s, 3H), 3.29 (dt, *J* = 11.6, 5.8 Hz, 1H), 3.07 – 2.94 (m, 1H), 2.80 – 2.69 (m, 1H), 2.26 – 2.11 (m, 2H), 2.11 – 1.94 (m, 1H), 1.30 (d, *J* = 6.5 Hz, 3H), 1.19 (d, *J* = 6.7 Hz, 3H). ^13^C NMR (126 MHz, CDCl_3_): δ 166.5, 166.3, 162.0, 153.1, 152.9, 147.3, 142.2, 140.8, 128.7, 126.2, 124.1, 124.0, 120.6, 117.3, 113.9, 112.3, 56.2, 46.9, 34.9, 31.1, 23.2, 22.4, 20.8, 20.3. LC-MS (ESI): *t*_R_ = 4.50 min, area: 96%, *m/z* 435 [M + H]^+^. HRMS (ESI) *m/z*: [M + H]^+^ calcd. for C_24_H_27_N_4_O_4_ 435.2027, found 435.2020.

- 1. *N*-(2-Amino-2-oxoethyl)-2-(5-(*cis*-3-isopropyl-4-oxo-3,4,4a,5,8,8a-hexahydrophthalazin-1-yl)-2-methoxyphenoxy)isonicotinamide (**3m**)

This compound was prepared from **3f** (140 mg, 0.321 mmol) and glycinamide∙HCl (42.6 mg, 0.386 mmol) as described for **3q**. The title compound was obtained as a white solid (120 mg, 76%).

^1^H NMR (500 MHz, CDCl_3_): δ 8.21 (d, *J* = 5.0 Hz, 1H), 7.69 (s, 1H), 7.66 (d, *J* = 8.6 Hz, 1H), 7.57 – 7.49 (m, 1H), 7.37 (s, 1H), 7.33 (d, *J* = 4.9 Hz, 1H), 7.01 (d, *J* = 8.6 Hz, 1H), 6.45 (s, 1H), 5.87 (s, 1H), 5.80 – 5.57 (m, 2H), 5.00 (hept, *J* = 6.4 Hz, 1H), 4.18 (d, *J* = 4.5 Hz, 2H), 3.76 (s, 3H), 3.27 (dt, *J* = 11.1, 5.3 Hz, 1H), 3.03 – 2.93 (m, 1H), 2.76 – 2.67 (m, 1H), 2.24 – 2.10 (m, 2H), 2.06 – 1.97 (m, 1H), 1.28 (d, *J* = 6.4 Hz, 3H), 1.16 (d, *J* = 6.6 Hz, 3H). ^13^C NMR (126 MHz, CDCl_3_): δ 170.8, 166.6, 165.7, 164.2, 153.1, 153.0, 148.5, 144.5, 142.3, 128.7, 126.1, 124.2, 124.1, 120.6, 115.7, 112.5, 108.9, 56.1, 46.8, 43.2, 34.8, 31.1, 23.2, 22.4, 20.7, 20.3. LC-MS (ESI): *t*_R_ = 3.85 min, area: >98%, *m/z* 492 [M + H]^+^. HRMS (ESI) *m/z*: [M + H]^+^ calcd. for C_26_H_30_N_5_O_5_ 492.2241, found 492.2224.

- 1. *N*-(2-Amino-2-oxoethyl)-6-(5-(*cis*-3-isopropyl-4-oxo-3,4,4a,5,8,8a-hexahydrophthalazin-1-yl)-2-methoxyphenoxy)nicotinamide (**3n**)

This compound was prepared from **3g** (150 mg, 0.344 mmol) and glycinamide∙HCl (762 mg, 6.89 mmol) as described for **3p**. The title compound was obtained after column chromatography using MeOH/DCM (gradient 0-10%) as a white solid (93 mg, 55%).

^1^H NMR (500 MHz, DMSO-*d*_6_): δ 8.78 (t, *J* = 5.9 Hz, 1H), 8.55 (d, *J* = 2.2 Hz, 1H), 8.25 (dd, *J* = 8.7, 2.4 Hz, 1H), 7.76 (dd, *J* = 8.7, 2.1 Hz, 1H), 7.69 (d, *J* = 2.1 Hz, 1H), 7.42 (s, 1H), 7.22 (d, *J* = 8.7 Hz, 1H), 7.11 (d, *J* = 8.6 Hz, 1H), 7.07 (s, 1H), 5.75 – 5.54 (m, 2H), 4.87 (hept, *J* = 6.6 Hz, 1H), 3.80 (d, *J* = 5.9 Hz, 2H), 3.73 (s, 3H), 3.45 (dt, *J* = 11.5, 5.7 Hz, 1H), 2.88 – 2.72 (m, 1H), 2.79 – 2.69 (m, 1H), 2.20 – 2.09 (m, 2H), 1.88 – 1.78 (m, 1H), 1.21 (d, *J* = 6.5 Hz, 3H), 1.12 (d, *J* = 6.7 Hz, 3H). ^13^C NMR (126 MHz, DMSO-*d*_6_): δ 170.9, 166.1, 164.5, 164.5, 153.1, 152.7, 147.2, 141.7, 139.2, 127.9, 125.9, 124.9, 124.4, 124.1, 120.2, 113.0, 109.8, 56.0, 45.8, 42.3, 33.9, 29.9, 22.6, 22.0, 20.5, 20.1. LC-MS (ESI): *t*_R_ = 3.90 min, area: 95%, *m/z* 488 [M + H]^+^. HRMS (ESI) *m/z*: [M + H]^+^ calcd. for C_26_H_30_N_5_O_5_ 492.2241, found 492.2226.

- 1. *N*-(2-Amino-2-oxoethyl)-6-(5-(*cis*-3-isopropyl-4-oxo-3,4,4a,5,8,8a-hexahydrophthalazin-1-yl)-2-methoxyphenoxy)picolinamide (**3o**)

This compound was prepared from **3h** (160 mg, 0.367 mmol) and glycinamide∙HCl (48.7 mg, 0.441 mmol) as described for **3q**. The title compound was obtained as a white solid (146 mg, 81%).

^1^H NMR (300 MHz, CDCl_3_): δ 7.99 (t, *J* = 5.5 Hz, 1H), 7.91 – 7.79 (m, 2H), 7.76 – 7.61 (m, 2H), 7.12 – 7.02 (m, 2H), 6.20 (s, 1H), 5.83 – 5.60 (m, 2H), 5.50 (s, 1H), 5.03 (hept, *J* = 7.0 Hz, 1H), 4.03 (d, *J* = 5.7 Hz, 2H), 3.81 (s, 3H), 3.31 (dt, *J* = 11.4, 5.2 Hz, 1H), 3.06 – 2.89 (m, 1H), 2.85 – 2.72 (m, 1H), 2.27 – 2.11 (m, 2H), 2.09 – 1.97 (m, 1H), 1.29 (d, *J* = 6.5 Hz, 3H), 1.19 (d, *J* = 6.7 Hz, 3H). ^13^C NMR (126 MHz, CDCl_3_): δ 170.9, 166.6, 164.7, 162.0, 153.1, 153.0, 146.9, 142.0, 140.8, 128.7, 126.2, 124.3, 124.0, 120.4, 117.2, 114.1, 112.4, 56.2, 46.8, 43.3, 34.8, 31.1, 23.3, 22.5, 20.8, 20.3. LC-MS (ESI): *t*_R_ = 4.19 min, area: 97%, *m/z* 492 [M + H]^+^. HRMS (ESI) *m/z*: [M + H]^+^ calcd. for C_26_H_30_N_5_O_5_ 492.2241, found 492.2228.

- 1. *N*-Butyl-2-(5-(*cis*-3-isopropyl-4-oxo-3,4,4a,5,8,8a-hexahydrophthalazin-1-yl)-2-methoxyphenoxy)nicotinamide (**3p**)

To a solution of **3e** (150 mg, 0.344 mmol) in DCM (3 mL) was added EDC∙HCl (198 mg, 1.03 mmol), HOBt∙H_2_O (158 mg, 1.03 mmol) and n-butylamine (0.69 mL, 7.0 mmol). The reaction mixture was stirred at rt for 72 h. The reaction mixture was diluted with DCM (25 mL) and washed with sat. aq. NH_4_Cl (2 × 25 mL) and sat. aq. NaHCO_3_ (2 × 25 mL). The organic phase was dried over MgSO_4_ and concentrated under reduced pressure to give a yellow oil. The product was purified by column chromatography using EtOAc/cyclohexane (gradient: 20-50%) to obtain the title compound as a white solid (86 mg, 50%)

^1^H NMR (500 MHz, DMSO-*d*_6_): δ 8.37 – 8.24 (m, 1H), 8.18 – 8.09 (m, 2H), 7.78 (s, 1H), 7.74 (d, *J* = 8.5 Hz, 1H), 7.22 – 7.15 (m, 2H), 5.74 – 5.59 (m, 2H), 4.87 (hept, *J* = 6.7 Hz, 1H), 3.73 (s, 3H), 3.47 – 3.40 (m, 1H), 3.34 – 3.28 (m, 2H), 2.81 (t, *J* = 5.0 Hz, 1H), 2.79 – 2.69 (m, 1H), 2.23 – 2.06 (m, 2H), 1.90 – 1.78 (m, 1H), 1.55 – 1.47 (m, 2H), 1.38 – 1.30 (m, 2H), 1.22 (d, *J* = 6.3 Hz, 3H), 1.13 (d, *J* = 6.6 Hz, 3H), 0.88 (t, *J* = 7.3 Hz, 3H). ^13^C NMR (126 MHz, CDCl_3_): δ 166.5, 163.7, 160.0, 152.8, 152.6, 149.5, 142.2, 141.6, 128.6, 126.1, 124.4, 124.0, 121.1, 119.4, 116.9, 112.2, 56.1, 46.8, 39.8, 34.8, 31.6, 31.1, 23.2, 22.4, 20.7, 20.3, 20.3, 13.9. LC-MS (ESI): *t*_R_ = 5.19 min, area: >98%, *m/z* 491 [M + H]^+^. HRMS (ESI) *m/z*: [M + H]^+^ calcd. for C_28_H_35_N_4_O_4_ 491.2653, found 491.2652.

- 1. *N*-Butyl-2-(5-(*cis*-3-isopropyl-4-oxo-3,4,4a,5,8,8a-hexahydrophthalazin-1-yl)-2-methoxyphenoxy)isonicotinamide (**3q**)

To a solution of **3f** (140 mg, 0.321 mmol) in DMF (2.3 mL) and DIPEA (0.23 mL, 1.3 mmol) was added HATU (0.15 g, 0.39 mmol) and n-butyl amine (0.1 mL, 1.0 mmol). The reaction mixture was stirred at rt for 24 h. The reaction mixture was diluted with DCM (30 mL) and washed with sat. aq. NH_4_Cl (30 mL). The aqueous phase was extracted with DCM (2 × 30 mL). The combined organic phases were washed with brine (2 × 80 mL), dried over Na_2_SO_4_ and concentrated under reduced pressure. The product was purified by column chromatography using EtOAc/cyclohexane (gradient 20-50%) to obtain the title compound as a white solid (91 mg, 58%).

^1^H NMR (500 MHz, CDCl_3_): δ 8.76 (t, *J* = 5.7 Hz, 1H), 8.18 (d, *J* = 5.2 Hz, 1H), 7.74 (dd, *J* = 8.6, 2.2 Hz, 1H), 7.67 (d, *J* = 2.1 Hz, 1H), 7.43 (dd, *J* = 5.2, 1.3 Hz, 1H), 7.36 (d, *J* = 1.1 Hz, 1H), 7.21 (d, *J* = 8.7 Hz, 1H), 5.73 – 5.57 (m, 2H), 4.85 (hept, *J* = 6.7 Hz, 1H), 3.73 (s, 3H), 3.49 – 3.41 (m, 1H), 3.27 (q, *J* = 6.9 Hz, 2H), 2.80 (t, *J* = 6.1 Hz, 1H), 2.77 – 2.69 (m, 1H), 2.20 – 2.07 (m, 2H), 1.88 – 1.77 (m, 1H), 1.56 – 1.46 (m, 2H), 1.38 – 1.28 (m, 2H), 1.21 (d, *J* = 6.5 Hz, 3H), 1.11 (d, *J* = 6.7 Hz, 3H), 0.90 (t, *J* = 7.3 Hz, 3H). ^13^C NMR (126 MHz, CDCl_3_): δ 171.2, 169.1, 168.7, 158.3, 158.0, 152.9, 150.8, 147.1, 133.1, 131.1, 129.4, 129.2, 125.4, 121.4, 118.1, 113.3, 61.1, 50.9, 39.0, 36.2, 35.1, 31.5, 27.8, 27.1, 25.6, 25.3, 24.8, 18.9. LC-MS (ESI): *t*_R_ = 4.87 min, area: 97%, *m/z* 491 [M + H]^+^. HRMS (ESI) *m/z*: [M + H]^+^ calcd. for C_28_H_35_N_4_O_4_ 491.2653, found 491.2632.

- 1. *N*-Butyl-6-(5-(*cis*-3-isopropyl-4-oxo-3,4,4a,5,8,8a-hexahydrophthalazin-1-yl)-2-methoxyphenoxy)nicotinamide (**3r**)

This compound was prepared from **3g** (150 mg, 0.344 mmol) and n-butylamine (0.69 mL, 7.0 mmol) as described for **3p**. The title compound was obtained as a white solid (47 mg, 28%).

^1^H NMR (300 MHz, CDCl_3_): δ 8.50 (d, *J* = 1.8 Hz, 1H), 8.13 (dd, *J* = 8.6, 2.3 Hz, 1H), 7.75 – 7.63 (m, 2H), 7.02 (t, *J* = 7.9 Hz, 2H), 6.05 (t, *J* = 4.9 Hz, 1H), 5.82 – 5.61 (m, 2H), 5.03 (hept, *J* = 6.5 Hz, 1H), 3.78 (s, 3H), 3.45 (q, *J* = 6.8 Hz, 2H), 3.28 (dt, *J* = 11.4, 5.8 Hz, 1H), 3.07 – 2.93 (m, 1H), 2.78 – 2.67 (m, 1H), 2.27 – 2.12 (m, 2H), 2.11 – 1.96 (m, 1H), 1.59 (p, *J* = 7.2 Hz, 2H), 1.47 – 1.33 (m, 2H), 1.29 (d, *J* = 6.5 Hz, 3H), 1.18 (d, *J* = 6.7 Hz, 3H), 0.95 (t, *J* = 7.3 Hz, 3H). ^13^C NMR (126 MHz, CDCl_3_): δ 166.5, 165.4, 165.2, 153.0, 152.9, 146.3, 142.2, 139.0, 128.8, 126.1, 125.6, 124.3, 124.1, 120.6, 112.5, 110.6, 56.2, 46.8, 40.0, 34.9, 31.8, 31.2, 23.2, 22.5, 20.7, 20.3, 20.3, 13.9. LC-MS (ESI): *t*_R_ = 4.75 min, area: 97%, *m/z* 547 [M + H]^+^. HRMS (ESI) *m/z*: [M + H]^+^ calcd. for C_28_H_35_N_4_O_4_ 491.2653, found 491.2634.

- 1. *N*-Butyl-6-(5-(*cis*-3-isopropyl-4-oxo-3,4,4a,5,8,8a-hexahydrophthalazin-1-yl)-2-methoxyphenoxy)picolinamide (**3s**)

This compound was prepared from **3h** (160 mg, 0.367 mmol) and *n*-butyl amine (0.10 mL, 1.0 mmol) as described for **3q**. The title compound was obtained as a white solid (129 mg, 72%).

^1^H NMR (500 MHz, CDCl_3_): δ 7.88 (d, *J* = 7.3 Hz, 1H), 7.82 (t, *J* = 7.8 Hz, 1H), 7.72 – 7.67 (m, 2H), 7.52 (t, *J* = 5.6 Hz, 1H), 7.04 (d, *J* = 9.2 Hz, 1H), 7.00 (d, *J* = 8.1 Hz, 1H), 5.81 – 5.63 (m, 2H), 5.03 (hept, *J* = 6.7 Hz, 1H), 3.79 (s, 3H), 3.33 (q, *J* = 6.8 Hz, 2H), 3.31 – 3.26 (m, 1H), 3.05 – 2.97 (m, 1H), 2.77 – 2.71 (m, 1H), 2.24 – 2.12 (m, 2H), 2.09 – 1.99 (m, 1H), 1.45 (p, *J* = 7.0 Hz, 2H), 1.30 (d, *J* = 6.6 Hz, 3H), 1.28 – 1.22 (m, 2H), 1.19 (d, *J* = 6.7 Hz, 3H), 0.88 (t, *J* = 7.4 Hz, 3H). ^13^C NMR (126 MHz, CDCl_3_): δ 166.5, 163.7, 161.9, 153.1, 152.9, 148.0, 142.2, 140.8, 128.7, 126.1, 124.1, 123.9, 120.7, 116.9, 112.9, 112.2, 56.1, 46.9, 38.9, 34.9, 31.5, 31.1, 23.2, 22.4, 20.7, 20.3, 20.1, 13.9. LC-MS (ESI): *t*_R_ = 5.23 min, area: >98%, *m/z* 491 [M + H]^+^. HRMS (ESI) *m/z*: [M + H]^+^ calcd. for C_28_H_35_N_4_O_4_ 491.2653, found 491.2629.

- 1. *N*-(Furan-2-ylmethyl)-2-(5-(*cis*-3-isopropyl-4-oxo-3,4,4a,5,8,8a-hexahydrophthalazin-1-yl)-2-methoxyphenoxy)nicotinamide (**3t**)

This compound was prepared from **3e** (150 mg, 0.344 mmol) and furfuryl amine (0.62 mL, 7.0 mmol) as described for **3i**. The title compound was obtained as a white solid (96 mg, 54%).

^1^H NMR (500 MHz, CDCl_3_): δ 8.81 (t, *J* = 5.6 Hz, 1H), 8.19 (d, *J* = 7.4 Hz, 1H), 8.16 (d, *J* = 4.7 Hz, 1H), 7.79 (s, 1H), 7.75 (d, *J* = 8.7 Hz, 1H), 7.58 (s, 1H), 7.21 (t, *J* = 6.2 Hz, 2H), 6.39 (s, 1H), 6.30 (s, 1H), 5.73 – 5.60 (m, 2H), 4.87 (hept, *J* = 6.1 Hz, 1H), 4.54 (d, *J* = 5.7 Hz, 2H), 3.71 (s, 3H), 3.43 (dt, *J* = 11.4, 5.6 Hz, 1H), 2.87– 2.75 (m, 1H), 2.78 – 2.70 (m, 1H), 2.21 – 2.08 (m, 2H), 1.89 – 1.78 (m, 1H), 1.39 (s, 2H), 1.22 (d, *J* = 6.5 Hz, 3H), 1.12 (d, *J* = 6.6 Hz, 3H). ^13^C NMR (126 MHz, DMSO-*d*_6_): δ 166.1, 163.7, 159.4, 153.2, 152.7, 152.2, 149.1, 142.2, 141.6, 140.6, 127.8, 125.9, 124.3, 124.1, 120.9, 118.9, 117.7, 112.8, 110.5, 106.8, 56.0, 45.8, 36.4, 33.9, 30.0, 22.6, 22.0, 20.5, 20.1. LC-MS (ESI): *t*_R_ = 4.96 min, area: >98%, *m/z* 515 [M + H]^+^. HRMS (ESI) *m/z*: [M + H]^+^ calcd. for C_29_H_31_N_4_O_5_ 515.2289, found 515.2290.

- 1. *N*-(Furan-2-ylmethyl)-2-(5-(*cis*-3-isopropyl-4-oxo-3,4,4a,5,8,8a-hexahydrophthalazin-1-yl)-2-methoxyphenoxy)isonicotinamide (**3u**)

This compound was prepared from **3f** (140 mg, 0.321 mmol) and furfuryl amine (0.10 mL, 1.1 mmol) as described for **3q**. The title compound was obtained as a white solid (102 mg, 62%). ^1^H NMR (500 MHz, CDCl_3_): δ 8.22 (d, *J* = 5.1 Hz, 1H), 7.69 (s, 1H), 7.66 (d, *J* = 8.6 Hz, 1H), 7.39 (s, 1H), 7.32 – 7.27 (m, 2H), 7.02 (d, *J* = 8.6 Hz, 1H), 6.62 (t, 1H), 6.34 (d, *J* = 15.6 Hz, 2H), 5.80 – 5.62 (m, 2H), 5.02 (hept, *J* = 6.2 Hz, 1H), 4.64 (d, *J* = 5.1 Hz, 2H), 3.77 (s, 3H), 3.27 (dt, *J* = 11.2, 5.5 Hz, 1H), 3.05 – 2.93 (m, 1H), 2.79 – 2.72 (m, 1H), 2.25 – 2.11 (m, 2H), 2.09 – 1.96 (m, 1H), 1.28 (d, *J* = 6.5 Hz, 3H), 1.17 (d, *J* = 6.6 Hz, 3H). ^13^C NMR (126 MHz, CDCl_3_): δ 166.6, 165.1, 164.2, 153.0, 153.0, 150.5, 148.5, 145.2, 142.7, 142.3, 128.7, 126.1, 124.2, 124.1, 120.6, 115.7, 112.5, 110.8, 108.7, 108.3, 56.2, 46.8, 37.2, 34.9, 31.1, 23.2, 22.5, 20.7, 20.3. LC-MS (ESI): *t*_R_ = 4.75 min, area: >98%, *m/z* 515 [M + H]^+^. HRMS (ESI) *m/z*: [M + H]^+^ calcd. for C_29_H_31_N_4_O_5_ 515.2289, found 515.2276.

- 1. *N*-(Furan-2-ylmethyl)-6-(5-(*cis*-3-isopropyl-4-oxo-3,4,4a,5,8,8a-hexahydrophthalazin-1-yl)-2-methoxyphenoxy)nicotinamide (**3v**)

This compound was prepared from **3g** (150 mg, 0.344 mmol) and furfuryl amine (0.61 mL, 6.9 mmol) as described for **3p**. The title compound was obtained as a white solid (54 mg, 31%).

^1^H NMR (500 MHz, CDCl_3_): δ 8.54 (s, 1H), 8.15 (d, *J* = 8.4 Hz, 1H), 7.74 – 7.65 (m, 2H), 7.37 (s, 1H), 7.02 (t, *J* = 9.1 Hz, 2H), 6.41 (s, 1H), 6.34 (s, 1H), 6.30 (s, 1H), 5.83 – 5.59 (m, 2H), 5.02 (hept, *J* = 6.3 Hz, 1H), 4.63 (d, *J* = 4.6 Hz, 2H), 3.77 (s, 3H), 3.41 – 3.27 (m, 1H), 3.12 – 2.87 (m, 1H), 2.74 – 2.68 (m, 1H), 2.28 – 2.11 (m, 2H), 2.10 – 1.97 (m, 1H), 1.29 (d, *J* = 6.3 Hz, 3H), 1.17 (d, *J* = 6.5 Hz, 3H).  ^13^C NMR (126 MHz, DMSO-*d*_6_): δ 166.1, 164.5, 164.2, 153.1, 152.7, 152.2, 147.1, 142.2, 141.7, 139.2, 128.0, 125.9, 124.9, 124.4, 124.1, 120.2, 113.0, 110.6, 109.9, 107.0, 56.0, 45.8, 36.0, 33.9, 29.9, 22.6, 22.0, 20.5, 20.1. LC-MS (ESI): *t*_R_ = 4.75 min, area: 98%, *m/z* 515 [M + H]^+^. HRMS (ESI) *m/z*: [M + H]^+^ calcd. for C_29_H_31_N_4_O_5_ 515.2289, found 515.2273.

- 1. *N*-(Furan-2-ylmethyl)-6-(5-(*cis*-3-isopropyl-4-oxo-3,4,4a,5,8,8a-hexahydrophthalazin-1-yl)-2-methoxyphenoxy)picolinamide (**3w**)

This compound was prepared from **3h** (160 mg, 0.367 mmol) and furfuryl amine (0.10 mL, 1.1 mmol) as described for **3q**. The title compound was obtained as a white solid (131 mg, 69%).

^1^H NMR (500 MHz, CDCl_3_): δ 7.92 – 7.89 (m, 1H), 7.85 – 7.81 (m, 1H), 7.79 (t, *J* = 5.7 Hz, 1H), 7.69 (d, *J* = 8.1 Hz, 2H), 7.32 (dd, *J* = 1.8, 0.7 Hz, 1H), 7.00 (s, 2H), 6.29 (dd, *J* = 3.2, 1.9 Hz, 1H), 6.18 – 6.13 (m, 1H), 5.81 – 5.62 (m, 2H), 5.04 (hept, *J* = 6.6 Hz, 1H), 4.52 (d, *J* = 5.7 Hz, 2H), 3.74 (s, 3H), 3.28 (dt, *J* = 11.5, 5.8 Hz, 1H), 3.05 – 2.97 (m, 1H), 2.77 – 2.65 (m, 1H), 2.24 – 2.11 (m, 2H), 2.07 – 1.97 (m, 1H), 1.30 (d, *J* = 6.6 Hz, 3H), 1.19 (d, *J* = 6.7 Hz, 3H).  ^13^C NMR (126 MHz, CDCl_3_): δ 166.5, 163.6, 161.9, 153.1, 152.9, 151.2, 147.6, 142.3, 142.1, 140.8, 128.7, 126.2, 124.1, 124.0, 120.5, 117.1, 113.2, 112.3, 110.5, 107.4, 56.0, 46.8, 36.5, 34.8, 31.1, 23.2, 22.4, 20.7, 20.3. LC-MS (ESI): *t*_R_ = 5.05 min, area: >98%, *m/z* 515 [M + H]^+^. HRMS (ESI) *m/z*: [M + H]^+^ calcd. for C_29_H_31_N_4_O_5_ 515.2289, found 515.2271.

# Appendix B: Virtual screening hits after visual inspection sorted by class.

Class 1: 5-membered aromatic heterocycles.

Class 2: 6-membered aromatic heterocycles

Class 3: Fused 5- and 6-membered aromatic heterocycles (5-ring bound)

Class 4: Fused 5- and 6-membered aromatic heterocycles (6-ring bound)

**3. Appendix C. Docking pose of selected hits for synthesis.**


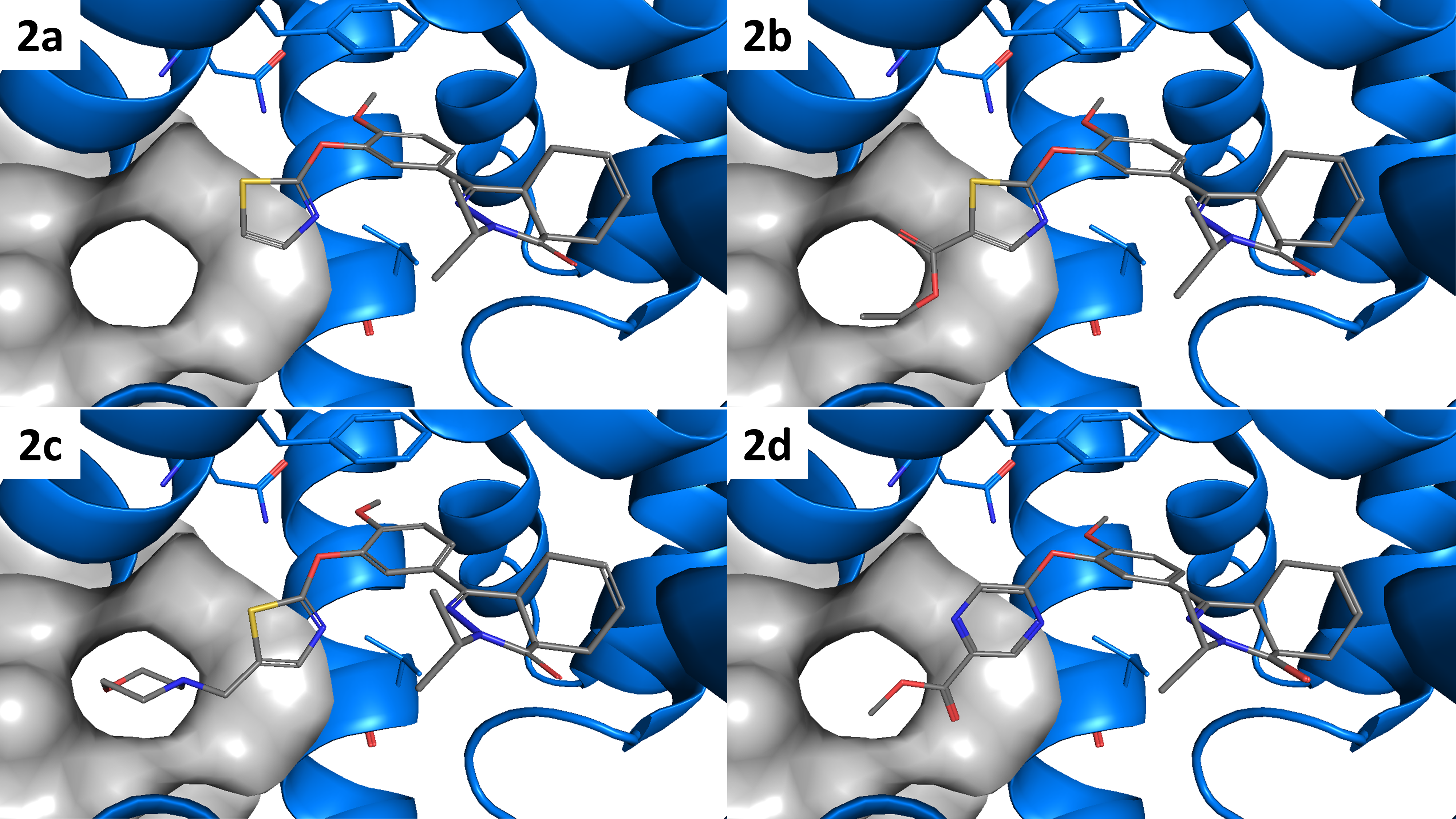

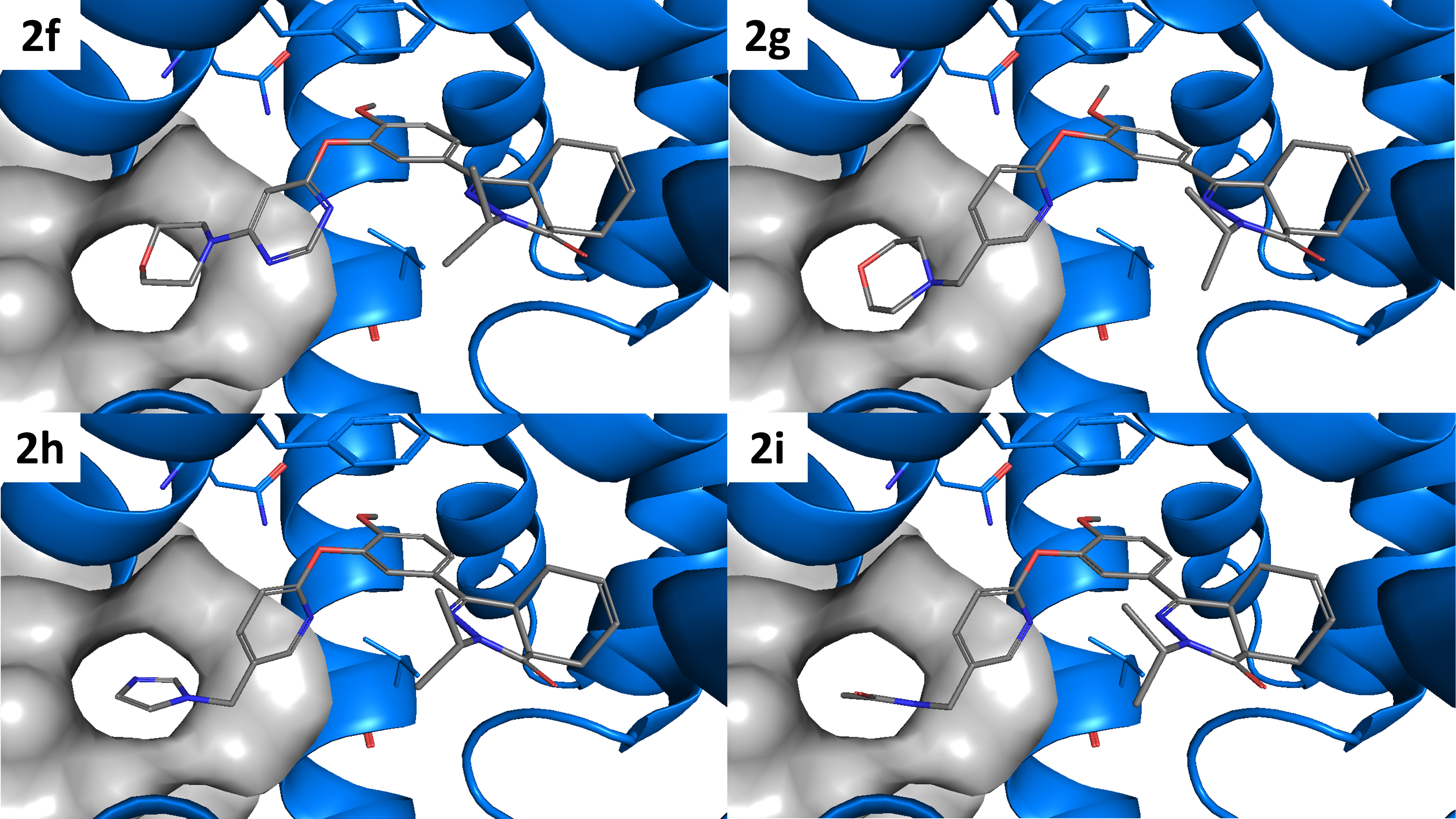

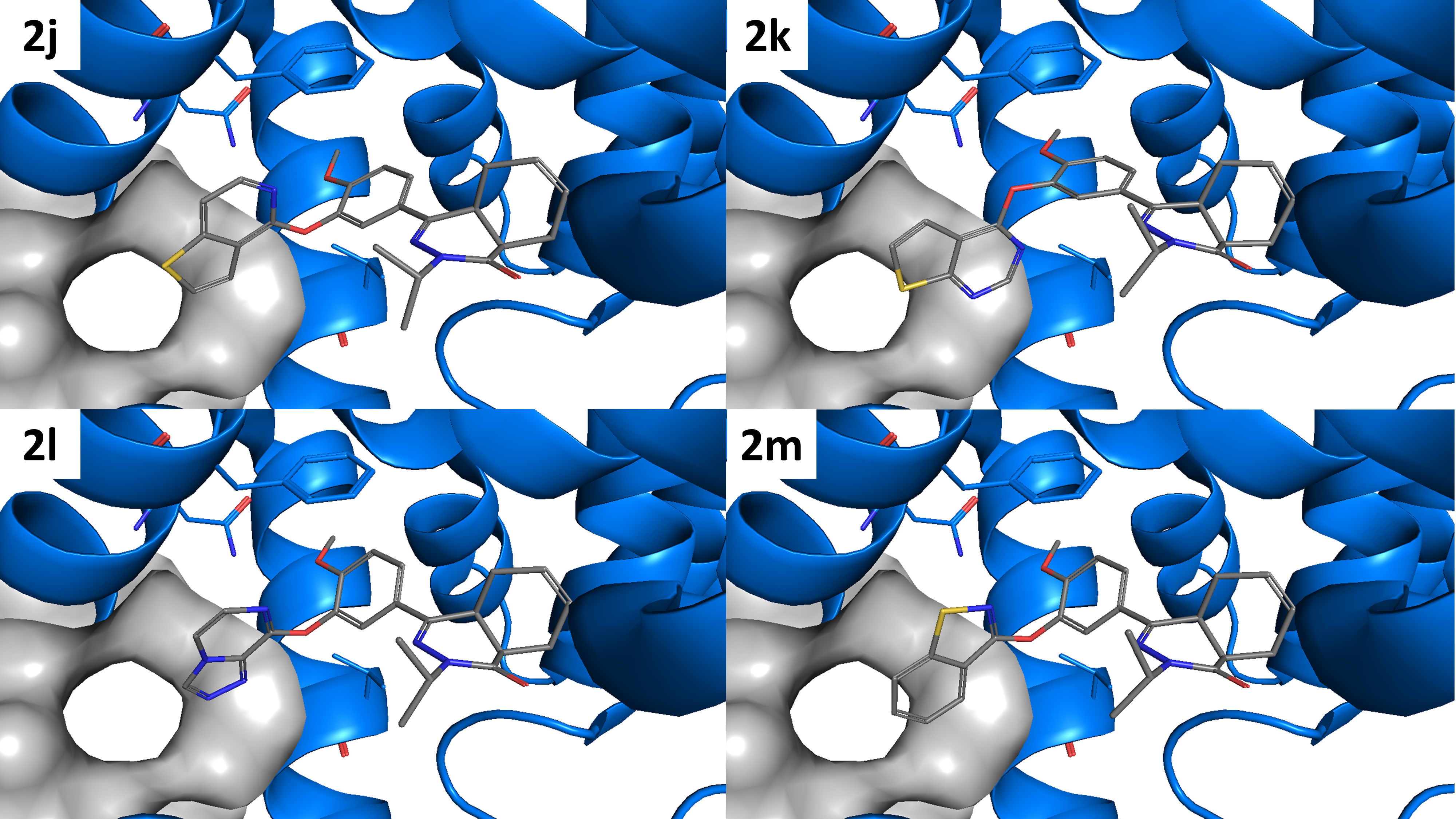

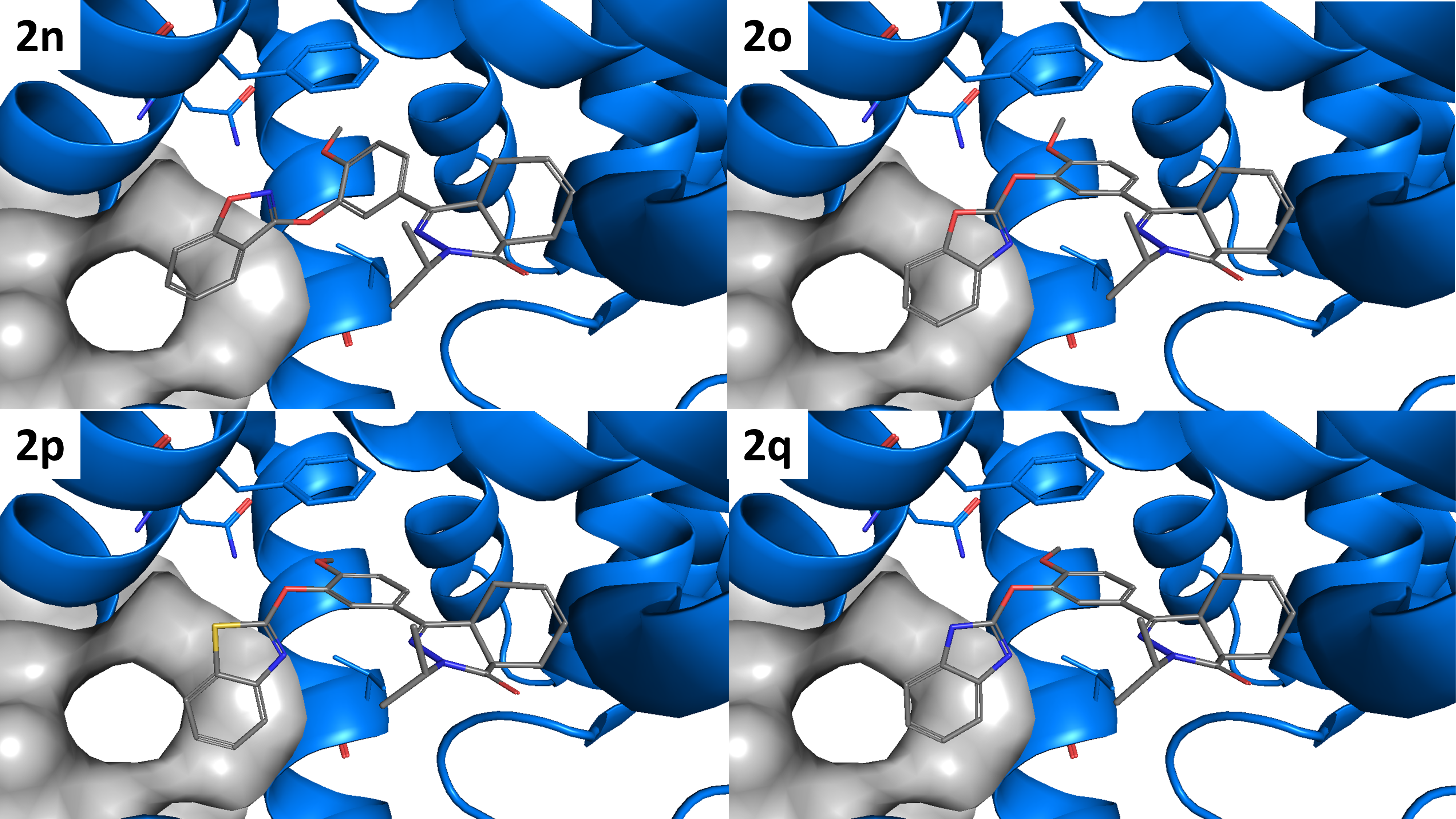

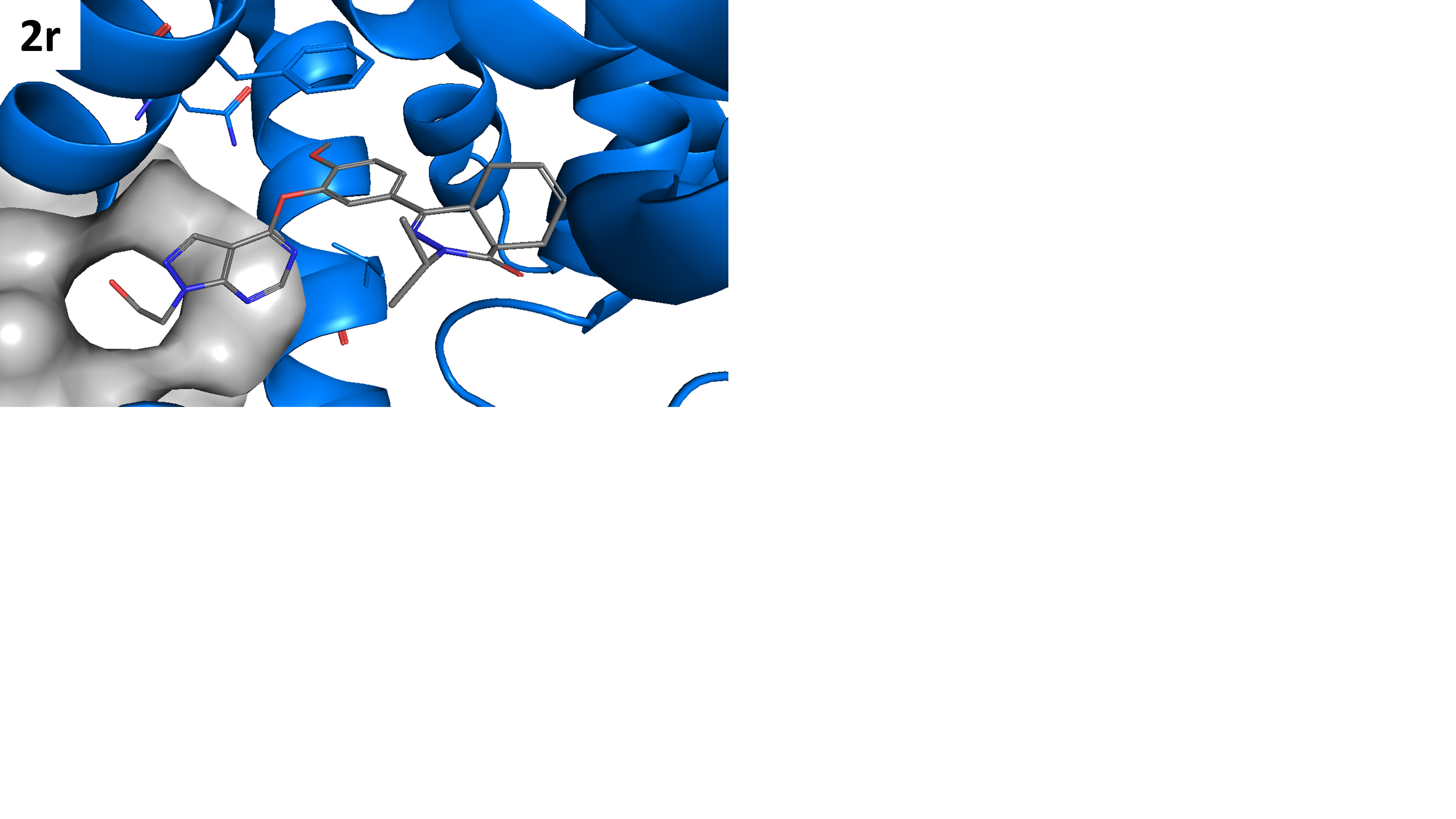

Supplement: Supplementary file 1 [file Table_1.DOCX]
